# To Dye or Not to Dye: Unraveling the Impact of Surface Chemistry on Cerium Oxide Nanoparticles–Cell Interactions

**DOI:** 10.1002/smsc.202500446

**Published:** 2025-12-18

**Authors:** Kanika Dulta, Thu Ngan Dinhová, Marie Hubálek Kalbáčová, Xiaohui Ju

**Affiliations:** ^1^ Department of Chemistry and Biochemistry Mendel University in Brno Zemědělská 1 Brno 61300 Czech Republic; ^2^ Center of Advanced Innovation Technologies VŠB‐Technical University of Ostrava Ostrava‐Poruba 70800 Czech Republic; ^3^ Department of Surface and Plasma Science Faculty of Mathematics and Physics Charles University v Holešovičkách 2 Prague 18000 Czech Republic; ^4^ Institute of Pathological Physiology 1st Faculty of Medicine Charles University U Nemocnice 5 Prague 12853 Czech Republic

**Keywords:** cellular uptake, cerium oxide, fluorescence, nanozyme, nanozyme‐cell interaction

## Abstract

Colloidally stable cerium oxide nanoparticles (CeNPs), known for mimicking multiple antioxidant enzymes, are promising nanozymes for therapeutic applications targeting oxidative stress and inflammation. Although fluorescent dye labeling facilitates nanoparticle imaging and tracking, its influence on physicochemical properties and biological interactions remains insufficiently understood. In this study, poly(acrylic acid)‐coated CeNPs and their DiI‐encapsulated counterparts are synthesized to evaluate the effects of dye functionalization on catalytic performance, cellular uptake, and intracellular fate. While overall redox cycling is retained, DiI@PAA‐CeNPs show an 30% decrease in superoxide dismutase‐like activity, whereas catalase‐, peroxidase‐, and oxidase‐like activities remain largely preserved. Both formulations exhibit no cytotoxicity toward human osteoblasts. However, DiI labeling modifies surface chemistry and delays cellular uptake, particularly in the presence of serum proteins, as revealed by fluorescence microscopy. Multiscale imaging combining fluorescence microscopy, transmission electron microscopy, and focused ion beam‐assisted lamella preparation reveals differences in nanoparticle internalization but similar intracellular fate. Focusing on poly(acrylic acid)‐coated cerium oxide nanoparticles, DiI encapsulation can markedly alter CeNPs properties. This finding highlights the need to critically assess labeling effects in bio‐nano studies. Integrating label‐free techniques is crucial for accurate characterization and the rational design of nanozymes for diagnostic and therapeutic applications.

## Graphical abstract

Fluorescent dye labeling enables nanoparticle imaging but may subtly alter their surface chemistry and biological behavior. This study compares dye‐labeled and unlabeled cerium oxide nanozymes, revealing changes in cellular uptake kinetics without affecting their final intracellular fate. Combined fluorescence and label‐free electron microscopy imaging provides multiscale insights essential for designing multifunctional nanozymes for therapeutic and diagnostic applications.

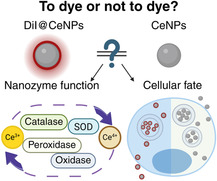

## 1 Introduction

Nanotechnology has revolutionized nanomedicine by enabling the design of nanoparticles (NPs) with unique physicochemical properties for biomedical applications, including imaging,^[^ ^1^, ^2^ ^]^ targeted drug delivery,^[^ ^3^, ^4^, ^5^ ^]^ biosensing,^[^ ^6^ ^]^ and innovative therapies such as antioxidant therapy.^[^ ^7^, ^8^ ^]^ Nanozyme is a category of nanomaterials that possess enzyme‐like activities. A recent review has pointed out that more than 550 nanomaterials exhibit peroxidase‐like activity, 40 exhibit catalase‐ or superoxide dismutase(SOD)‐like activity, and 70 exhibit oxidase‐like activity, including materials such as fullerenes (C_60_) and noble metal‐based NPs.^[^ ^9^ ^]^ With the rapid development and ever‐deepening understanding of nanotechnology, nanozymes offer higher catalytic stability, ease of modification, and lower manufacturing cost than protein‐based enzymes.^[^ ^10^ ^]^ Among these, cerium oxide NPs (CeNPs), otherwise known as nanoceria, have gained particular attention as nanozymes due to their distinctive redox cycling between Ce^3+^ and Ce^4+^ oxidation states, enabling them with multienzyme mimetic properties to scavenge reactive oxygen species (ROS). This redox versatility, enhanced by available oxygen vacancies on the surface of the NPs, underpins their remarkable ROS‐scavenging capacity and broad application in areas ranging from catalysis to antioxidant nanomedicine.^[^ ^11^ ^]^ CeNPs can mimic several key endogenous antioxidant enzymes, such as SOD, peroxidase, catalase, and oxidase, facilitating the efficient scavenging of endogenous ROS such as superoxide anions, hydrogen peroxide, and peroxynitrite.^[^ ^12^, ^13^ ^]^

Despite their therapeutic potential, the clinical translation of CeNPs remains limited by challenges such as maintaining their colloidal stability and tracking their cellular fate in biological settings. To address these limitations, surface functionalization strategies have been employed to improve their dispersibility, mitigate cytotoxic effects, and maintain antioxidant performance across a range of pH environments.^[^ ^14^, ^15^ ^]^ These surface‐engineered CeNPs demonstrate promising potential in preclinical models of neurodegenerative disorders, inflammation, and radiation‐induced damage.^[^ ^16^, ^17^ ^]^ Nevertheless, their nanoscale dimensions raise critical concerns regarding unwanted interaction with biomacromolecules, including proteins, nucleic acids, and lipids, which may elicit adverse toxicological responses.^[^ ^18^, ^19^ ^]^ Such responses are highly dependent on physicochemical factors such as CeNPs surface morphology, cerium oxidation state, and capping agents.^[^ ^20^ ^]^ The interactions at the nanozyme–biological interface have often been underestimated, as nanozymes can undergo significant transformations upon entering physiological environments, resulting in a biological identity that differs markedly from their original synthetic form.^[^ ^21^, ^22^ ^]^

Moreover, recent studies have highlighted the critical role of chemical structure in determining nanomedicine safety and efficacy, emphasizing the need to explore the chemical design space to optimize their performance, including precise therapeutic component positioning for improved target engagement and programed carrier interactions for cell‐specific, triggered drug release.^[^ ^23^ ^]^ To date, numerical studies indicate that cellular uptake of NPs is a complicated process that includes phagocytosis, endocytosis, and direct transmembrane transport, which is governed by multiple factors.^[^ ^24^, ^25^, ^26^ ^]^ For instance, the physicochemical properties (such as size, shape, rigidity, and surface charges) of NPs can greatly affect their uptake efficiency and corresponding pathways.^[^ ^21^ ^]^ Different types of surface decorations (including nonspecific polymers and specific ligands) have also been widely used to modify the physicochemical properties of bare NPs.^[^ ^27^ ^]^ Surface charge is a critical determinant of nanozyme‐cell interactions, significantly influencing subsequent cellular uptake and intracellular trafficking.^[^ ^28^ ^]^ Asati et al. demonstrated that positively or negatively charged nanoceria showed lysosomal accumulation and varying toxicity profiles, while neutral dextran‐coated CeNPs exhibit minimal toxicity and diffuse cytoplasmic distribution.^[^ ^29^ ^]^

Fluorescent labeling is a widely used strategy to visualize the cellular fate of nanozymes and quantify their uptake, serving as an essential tool for investigating nanozyme‐cell interactions by traditional approaches (microscopic and spectroscopic methods). While these methods assume a linear relationship between fluorescence intensity and nanoparticle quantity, they can be flawed due to dye quenching at high concentrations or dye leaching due to nonspecific adsorptions.^[^ ^30^, ^31^ ^]^ Nevertheless, when carefully optimized, fluorescent labeling enables real‐time, noninvasive tracking of nanozymes in the cells. Two main fluorescent labeling strategies are often employed: 1) a dye encapsulation within the nanoparticle‐polymer coating matrix, or 2) a dye adsorption onto the surface, with the choice largely depending on dye hydrophobicity.^[^ ^32^, ^33^ ^]^ Hydrophobic dyes are typically encapsulated via hydrophobic‐hydrophilic interactions with polymeric NPs or liposomes,^[^ ^34^, ^35^ ^]^ while hydrophilic dyes are usually surface‐adsorbed to inorganic NPs or incorporated into their porous structures.^[^ ^36^, ^37^ ^]^ Fluorescent labeling of NPs is essential for their visualization for in vitro and in vivo experiments; however, the impact of this staining on the NPs’ physicochemical properties and subsequent biological interactions is rarely examined.

As previously mentioned, even subtle changes in surface chemistry can significantly influence nanozyme behavior in biological environments, potentially confounding experimental outcomes. Therefore, we aim to address this often‐overlooked question:^[^ ^38^, ^39^ ^]^ “to dye or not to dye”—what is the impact of fluorescent labeling on nanozyme‐cell interactions and their cellular fate? In this study, we used well‐characterized polymer‐coated cerium oxide NPs (PAA‐CeNPs) as our model system,^[^ ^40^ ^]^ and compared unlabeled NPs with fluorescently labeled NPs using the lipophilic dye DiI to produce DiI@PAA‐CeNPs. We comprehensively characterized their physicochemical properties and investigated their interaction with biological systems through (1) structural and optical characterization of NPs; (2) cellular uptake and internalization kinetics; (3) intracellular localization via their fluorescence characteristics; and (4) direct observation of particles via electron microscopy. By examining how dye functionalization affects the CeNPs‐based nanozyme's physicochemical properties, redox activity, and cellular interactions, we establish a reliable platform for evaluating the role of surface fluorescent dyes in nanozyme uptake and bio‐interactions, ultimately enabling more accurate interpretation in future studies. CeNPs retained their catalytic activity and biocompatibility as potential nanozymes after DiI incorporation. While no major differences in cellular fate were observed, DiI‐labeled CeNPs showed a slight delay in cellular internalization, likely due to interactions with serum proteins. These results highlight the critical importance of even subtle modifications at the nano–bio interface in shaping nanoparticle behavior within biological environments. Such insights are essential for the rational design of functional nanomaterials for biomedical applications.

## 2 Results and Discussion

### 2.1 Physicochemical Characterization and Comparison of PAA‐CeNPs and DiI@PAA‐CeNPs

**Figure 1:**
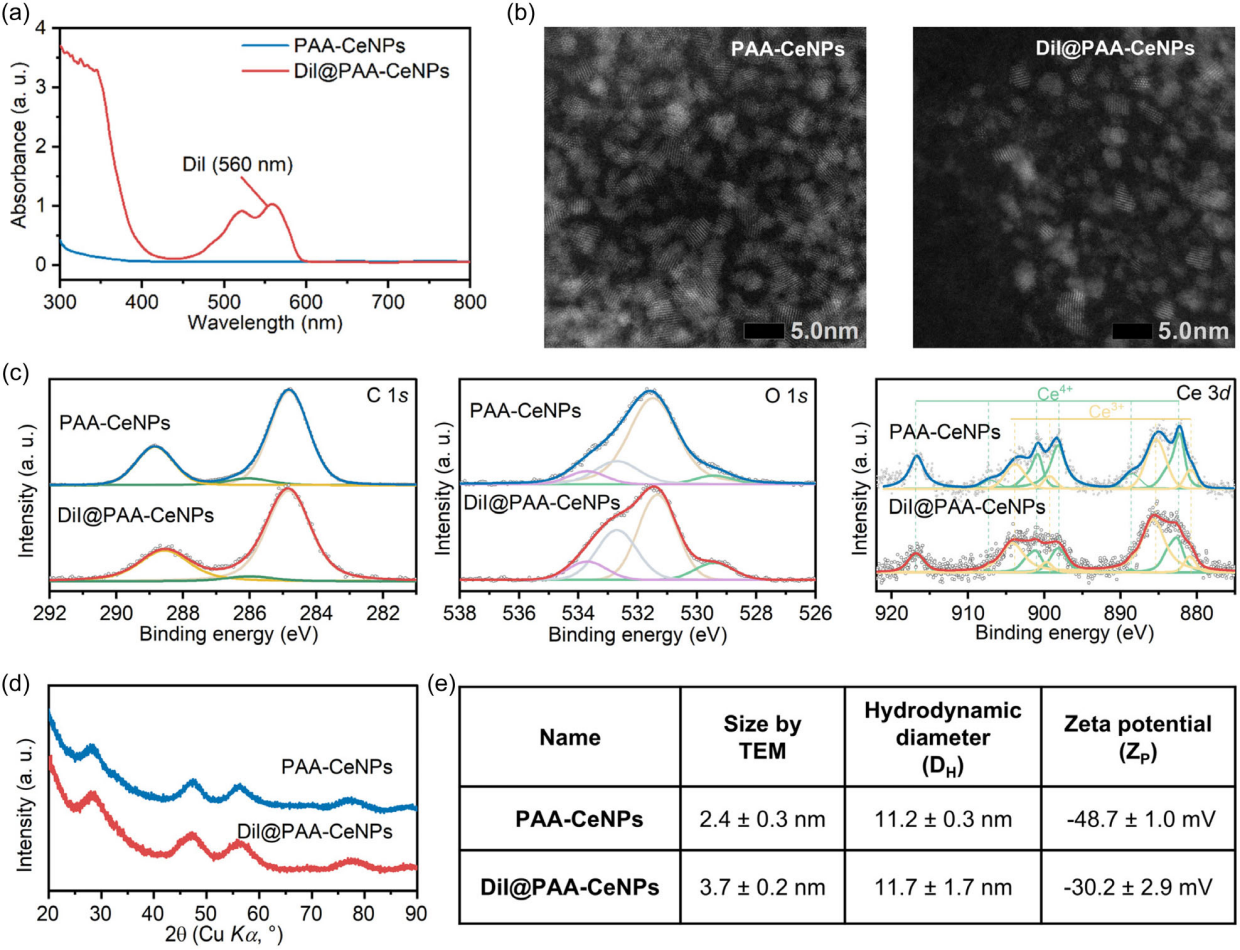
Physicochemical characterization and comparison between PAA‐CeNPs and DiI@PAA‐CeNPs. a) UV–Vis absorption spectra of PAA‐CeNPs and DiI@PAA‐CeNPs, both at a concentration of 1 mg ml^−1^. b) TEM micrographs of PAA‐CeNPs and DiI@PAA‐CeNPs. c) XPS core‐level spectra of Ce 3*d*, C 1*s,* and O 1*s* regions for PAA‐CeNPs (top) and DiI@PAA‐CeNPs (bottom). d) XRD patterns of PAA‐CeNPs (top) and DiI@PAA‐CeNPs (bottom). e) Comparison of size measured by TEM, hydrodynamic diameter measured by DLS, and zeta potential of PAA‐CeNPs and DiI@PAA‐CeNPs in water at 0.1 mg ml^−1^.

Firstly, we utilized the wet‐chemical precipitation method to synthesize the near‐spherical CeNPs coated with PAA.^[^ ^40^, ^41^ ^]^ These PAA‐CeNPs were subsequently subjected to fluorescent dye encapsulation. The lipophilic fluorescent dye DiI (1,1′‐dioctadecyl‐3,3,3′,3′‐tetramethylindocarbocyanine perchlorate) is a membrane stain based on carbocyanine that has been in extensive use since the early 1980s. It has a hydrophilic head group that associates with the membrane surface, along with two long alkyl chains that are inserted into the hydrophobic core of lipid bilayers, thus facilitating stable and efficient cellular membrane labeling.^[^ ^42^, ^43^ ^]^ Successful loading of DiI dye onto PAA‐CeNPs, resulting in DiI@PAA‐CeNPs, was confirmed via UV–Vis spectroscopy as shown in **Figure** **1**a. The characteristic absorbance peak at 560 nm is attributed to the DiI, confirming the effective binding of DiI to the PAA‐CeNPs. Transmission electron microscopy (TEM) was employed to evaluate the size and morphology of the PAA‐CeNPs and DiI@PAA‐CeNPs. High‐resolution TEM images in Figure 1b reveal that PAA‐CeNPs have quasi‐spherical crystallites with clearly resolved lattice fringes, which are characteristic of the fluorite CeO_2_ phase. Quantitative analysis of over 200 individual NPs indicates an average crystal diameter of 2.4 nm ± 0.3 nm. In the case of DiI@PAA‐CeNPs, the NPs remained uniformly dispersed and retained well‐defined lattice fringes despite the presence of the organic dye during synthesis. Statistical evaluation of these particles yields an average diameter of 3.7 nm ± 0.2 nm. These observations confirm that the incorporation of DiI did not compromise the crystalline structure and size of PAA‐CeNPs.

X‐ray photoelectron spectroscopy (XPS) was used to probe the surface chemistry of PAA‐CeNPs and DiI@PAA‐CeNPs, focusing on both the cerium oxidation state and the nature of surface ligands (Figure 1c). As shown in the Ce 3*d* core‐level spectra, PAA‐CeNPs exhibit a mixture of Ce^4+^ and Ce^3+^ species, with Ce^3+^ constituting ≈22% of the total oxidation state. Encapsulation of the fluorescent dye DiI increased the Ce^3+^ fraction to ≈39%, indicating that the DiI functionalization resulted in a higher reduced level of CeNPs. This increase in the Ce^3+^ fraction is unexpected, as the influence of dye incorporation on the surface oxidation state of CeNPs has not been investigated in the literature. However, we have observed previously that the adsorption of organic moieties onto cerium oxide surfaces can facilitate electron charge transfer between the adsorbed species and the oxygen vacancies on cerium oxide surfaces.^[^ ^44^ ^]^ We therefore hypothesized that the observed increase in Ce^3+^ fraction is primarily due to electron charge transfer facilitated by the physicochemical adsorption of the organic dyes onto the cerium oxide surface.

The C 1*s* spectra of PAA‐CeNPs show three distinct peaks at 284.8 eV, 286.4 eV, and 289.0 eV, which can be assigned to C‐C/C‐H (alkyl backbone of PAA), C‐O or C‐COOH (hydroxyl‐bearing carbons), and O‐C=O (carboxylate/carboxylic acid moieties), respectively. These features confirm the presence of PAA bound to the NPs surface as previously reported.^[^ ^45^ ^]^ After DiI encapsulation, the C 1*s* envelope broadened slightly, with peaks appearing at ≈284.8 eV (C‐C/C‐H), 286.7 eV (C‐O or ether linkages introduced by DiI), and 288.9 eV (HO‐C=O or esterified/carboxylic groups). The peak shift from 286.4 eV to 286.7 eV and the appearance of a shoulder at 288.9 eV signify that DiI's aromatic and alkyl chains, which contain additional oxygenated linkages, have successfully grafted onto the PAA layer. Analysis of the O 1*s* spectra further supports this interpretation, where three peaks were fitted for PAA‐CeNPs: a dominant peak at 529.3 eV, characteristic of lattice oxygen in the CeO_2_ fluorite structure; a second peak at 531.4 eV, attributed to surface hydroxyls; and a third component at 532.9 eV, arising from carboxylate oxygen (O=C‐OH or O‐C=O) within the PAA coating. In contrast, the O 1*s* spectra of DiI@PAA‐CeNPs consist of four components: the lattice oxygen peak remains at 529.3 eV, while an increased hydroxyl/defect‐related feature appears at 530.9 eV. Two additional peaks at 532.1 eV and 533.9 eV correspond to ester or C=O groups (likely stemming from DiI's oxygenated functional groups) and carboxylic acids or esterified PAA, respectively. The higher intensity in the 530–534 eV region reflects an enriched population of oxygenated species on the nanoparticle surface after DiI encapsulation. Together, these XPS results demonstrate that PAA functionalization and subsequent DiI attachment significantly modified the CeNPs surface, not only by increasing the Ce^3+^ fraction and oxygen‐vacancy density, but also by introducing new carbon‐ and oxygen‐containing functionalities. These changes in surface chemistry are likely to influence both the NPs’ reactivity and their interactions with surrounding media.

Powder X‐ray diffraction (XRD) analysis was performed to elucidate the crystallographic structure and average crystallite dimensions before and after DiI incorporation. As shown in Figure 1d, both PAA‐CeNPs and DiI@PAA‐CeNPs exhibit a series of broadened diffraction maxima centered at 2*θ* values of ≈28.5°, 33.0°, 47.5°, 56.3°, and 59.0°, which can be indexed to the (111), (200), (220), (311), and (222) reflections of the fluorite‐type CeO_2_ lattice (space group Fm‐3m). The absence of additional peaks confirms that the incorporation of PAA as well as DiI does not alter the crystal structure of cerium oxide. The Scherrer equation was applied to estimate the average crystallite size from the most intense (111) reflection at ≈28.5°. Analysis of the (111) full‐width at half‐maximum yields crystallite dimensions in the range of ≈1.7 to 2.2 nm, corresponding to the TEM observations. TEM measurements report the overall particle size, including the amorphous PAA‐DiI coating, whereas XRD reflects the size of the crystalline CeO_2_ core (≈2 nm). The larger particle dimensions observed by TEM after labeling are thus due to the organic shell rather than changes in the inorganic crystallites. Notably, the overlaid diffractograms of PAA‐CeNPs and DiI@PAA‐CeNPs reveal identical peak positions and comparable peak widths, indicating that the presence of the DiI dye does not induce changes in the crystallite parameters.

The number‐weighted hydrodynamic diameters (*D*_H_) of PAA‐CeNPs and DiI@PAA‐CeNPs were determined to be 11.2 ± 0.3 nm and 11.7 ± 1.7 nm, respectively, by dynamic light scattering (DLS). Representative DLS distribution of PAA‐CeNPs was shown in Figure S3, Supporting Information. This result confirms that both formulations contain uniformly dispersed colloidal particles. Zeta potential measurements reveal a decrease in negative surface charge from −48.7 ± 1.0 mV for PAA‐CeNPs to −30.2 ± 2.9 mV for DiI@PAA‐CeNPs. Although the decrease in the absolute value of zeta potential may be attributed to partial occupation of anionic binding sites by the dye since DiI is a cationic (positively charged) lipophilic dye, the value remains sufficiently large enough (absolute value over 20 mV) to maintain electrostatic stabilization of the colloidal particles in aqueous media.^[^ ^46^ ^]^ Consequently, these DLS and zeta potential results demonstrate that DiI loading does not compromise colloidal stability or particle size uniformity. These findings align with our previous reports.^[^ ^40^ ^]^ Previous research also reported that indocyanine green dye‐loaded NPs in the size around 100 nm remained stable over time with minimal changes in size, fluorescence, surface charge, or structure, indicating strong dye integration and enhanced functional properties.^[^ ^47^ ^]^

### 2.2 Colloidal Stability and Antioxidant Activity of PAA‐CeNPs and DiI@PAA‐CeNPs in Biological Media

**Figure 2:**
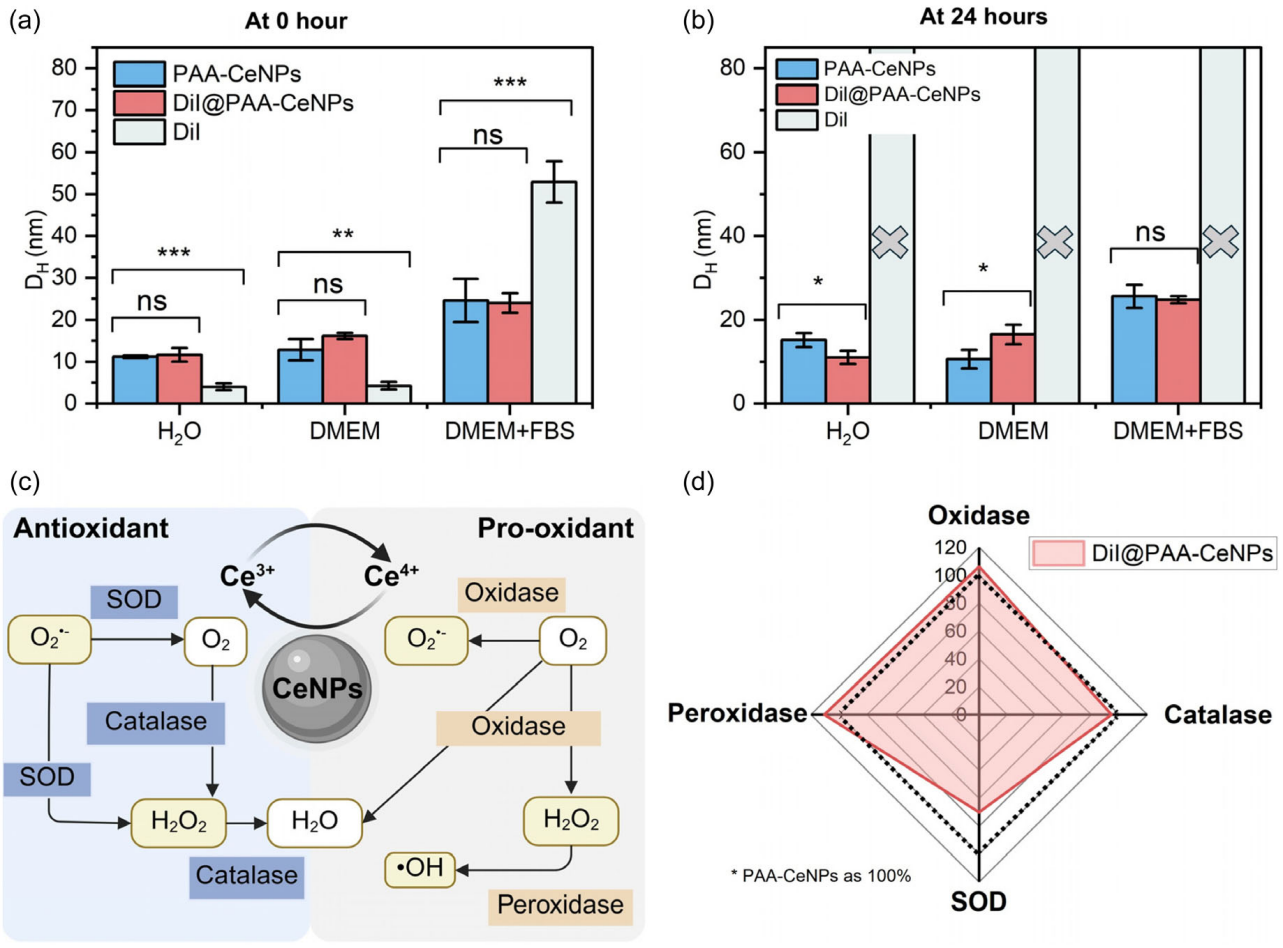
Colloidal stability and antioxidant activity of dye‐encapsulated PAA‐CeNPs. a) Hydrodynamic diameters of PAA‐CeNPs and DiI@PAA‐CeNPs measured at 0 h in water, DMEM cell culture medium, and DMEM supplemented with fetal bovine serum (FBS). b) Hydrodynamic diameters of PAA‐CeNPs and DiI@PAA‐CeNPs after 24 h of incubation under the same media conditions. c) Schematic illustration of the ROS modulation mechanism by CeNPs, which mimic multiple antioxidant and pro‐oxidant enzymes—including superoxide dismutase (SOD), catalase, peroxidase, and oxidase—through redox switching between Ce^3+^ and Ce^4+^ states. Schematic created with BioRender.com. d) Radar chart comparing the enzyme‐mimetic activities of DiI@PAA‐CeNPs, normalized to 100% activity of PAA‐CeNPs. Statistical analysis was performed using one‐way ANOVA; ns represents no significant difference (*P* > 0.05), and asterisks indicate statistical significance: ^*^ *P* < 0.05, ^**^ *P* < 0.01, and ^***^ *P* < 0.001.

A major concern in applying CeNPs nanozymes in biomedical settings lies in preserving their colloidal stability and enzyme‐like catalytic functions once they are introduced into the cellular environment. Therefore, we assessed how fluorescent labeling affects the surface characteristics of CeNPs, particularly in terms of their stability and catalytic performance in cell culture media before cellular uptake. As shown in **Figure** **2**a, both PAA‐CeNPs and DiI@PAA‐CeNPs exhibit very limited increases in hydrodynamic size when dispersed in cell culture media, Dulbecco's Modified Eagle Medium (DMEM), with or without fetal bovine serum (FBS) supplementation. FBS is commonly added to mimic physiological conditions, as it contains a variety of proteins and biomolecules. Notably, FBS may interact with nanoparticle surfaces, potentially altering their colloidal behavior and biological activity.^[^ ^48^, ^49^ ^]^ Our current observation confirms minimal media‐induced aggregation for both particles, consistent with our previous findings.^[^ ^40^ ^]^ Comparatively, there was no significant difference in the colloidal stability of PAA‐CeNPs and DiI@PAA‐CeNPs in both media settings (with or without FBS), suggesting that dye loading does not substantially impact nanozyme colloidal stability in the cell culture media. When monitored over a longer course, particle hydrodynamic sizes remained consistent over 24 h, indicating good temporal stability (Figure 2b). In contrast, DiI alone showed poor stability in biological media. In DMEM with FBS, DiI formed agglomerates immediately upon dispersion. In DMEM without FBS, DiI was only transiently stable, with significant agglomeration occurring within a few hours. These observations highlight the potential for free dye interactions with media components, especially proteins in FBS, to influence their colloidal behavior. Therefore, the interaction between nonadsorbed DiI and biological media with protein‐rich serum should be carefully considered when evaluating nanoparticle stability and their behavior in vitro and in vivo.

As shown in Figure 2c, the schematic depiction illustrates a dynamic, self‐regulating mechanism by which CeNPs‐based nanozyme modulates ROS species through reversible cycling between Ce^3+^ and Ce^4+^ oxidation states, exhibiting pro‐oxidant and antioxidant activities.^[^ ^50^, ^51^ ^]^ Central to this process is the facile conversion of cerium ions at oxygen vacancy sites on the surface, which enables conditional engagement of SOD‐, catalase‐, peroxidase‐, and oxidase‐like activities in response to the redox environment. Specifically, under conditions of elevated superoxide concentration, surface Ce^3+^ sites donate electrons to superoxide anions (O_2_^•−^), thereby oxidizing Ce^3+^ to Ce^4+^ while concomitantly generating molecular oxygen. A subsequent interaction between a second superoxide molecule and the newly formed Ce^4+^ yields hydrogen peroxide (H_2_O_2_) and regenerates Ce^3+^, effecting an SOD‐mimetic dismutation that attenuates the accumulation of the highly reactive superoxide radical. On the other hand, CeNPs can also exhibit pro‐oxidant‐like activities. Following H_2_O_2_ production, the Ce^4+^ enriched surface transitions to a catalase‐like reaction, decomposing H_2_O_2_ into water and oxygen without requiring external reducing equivalents. The availability of Ce^4+^ for this reaction scavenges excessive H_2_O_2_ into subcellular compartments where it might otherwise participate in Fenton‐type chemistry to form hydroxyl radicals (^•^OH). In environments rich in both H_2_O_2_ and reducing substrates, Ce^3+^ species facilitate peroxidase‐like activity by participating in redox cycling: Ce^3+^ is oxidized to Ce^4+^, enabling the reduction of H_2_O_2_ and concurrent substrates oxidation. Conversely, under conditions of limited reductant availability but plentiful molecular oxygen, Ce^4+^ exhibits oxidase‐like behavior by oxidizing susceptible biomolecules. Crucially, these enzymatic mimetic pathways are interdependent and continuously self‐regulating: Ce^3+^ and Ce^4+^ coexist on the nanoparticle surface, enabling simultaneous or sequential engagement of SOD‐, catalase‐, peroxidase‐, and oxidase‐like reactions as dictated by local ROS concentrations and reductant availability.

We investigated whether the incorporation of the fluorescent dye with subsequent changes of Ce^3+^ fractions influenced the multienzyme mimicking activities. Catalytic activities of these synthesized CeNPs nanozymes as redox enzyme mimetics were tested via colorimetric‐based activity assay. As shown in Figure 2d, the radar chart comparing DiI@PAA‐CeNPs to PAA‐CeNPs (normalized to 100% as indicated by the dashed line) reveals that dye loading has a differential impact on each enzyme‐mimetic activity relative to the original PAA‐CeNPs. Specifically, DiI@PAA‐CeNPs exhibit rather significant diminished SOD‐like activity (≈70% of the PAA‐CeNPs), indicating that incorporated DiI may alter Ce^3+^ sites responsible for superoxide dismutation, although the dye increased the apparent Ce^3+^ fractions. In contrast, catalase‐like activity is largely preserved upon dye encapsulation, measuring ≈95% of the unmodified PAA‐CeNPs value; this suggests that the H_2_O_2_‐decomposing Ce^4+^ sites remain accessible and catalytically competent. Peroxidase‐ and oxidase‐like activities were minimally affected by DiI functionalization (≈110% and ≈105%, respectively), implying that slight electronic or structural modifications introduced by PAA‐DiI interaction may facilitate electron transfer during H_2_O_2_ reduction (peroxidase) and O_2_ oxidation (oxidase). Taken together, these findings demonstrate that, although incorporation of the lipophilic dye diminishes SOD‐mimetic turnover, the overall redox‐cycling capacity of CeNPs is retained—and in some cases improved (pro‐oxidant)—after DiI loading, thereby preserving their multifaceted nanozyme potential. Research has indicated that the Ce^3+^ fraction is the most critical factor for CeNPs’ antioxidant activities.^[^ ^40^, ^52^, ^53^ ^]^ Our findings suggest that the changes in the Ce^3+^/Ce^4+^ ratio induced by dye incorporation slightly reduced their antioxidant enzymatic activity. The presence of organic molecules appears to partially hinder their SOD‐like activity, although the Ce^3+^ fractions were increased. However, we currently do not have additional experimental data to directly confirm this mechanism or support another hypothesis. Therefore, this proposed explanation should be considered a plausible interpretation supported by indirect evidence, and further studies would be valuable to quantitatively validate the impact of dye encapsulation on nanozyme activity. These findings also indicate that establishing a strictly linear relationship between the Ce^3+^ fractions and antioxidant activity is challenging, as numerous factors can influence the surface properties of CeNPs.^[^ ^54^ ^]^ This complexity will become even more pronounced once the NPs are introduced into biological environments.

### 2.3 Cytotoxicity Evaluation and Cell Interaction with PAA‐CeNPs and DiI@PAA‐CeNPs

**Figure 3:**
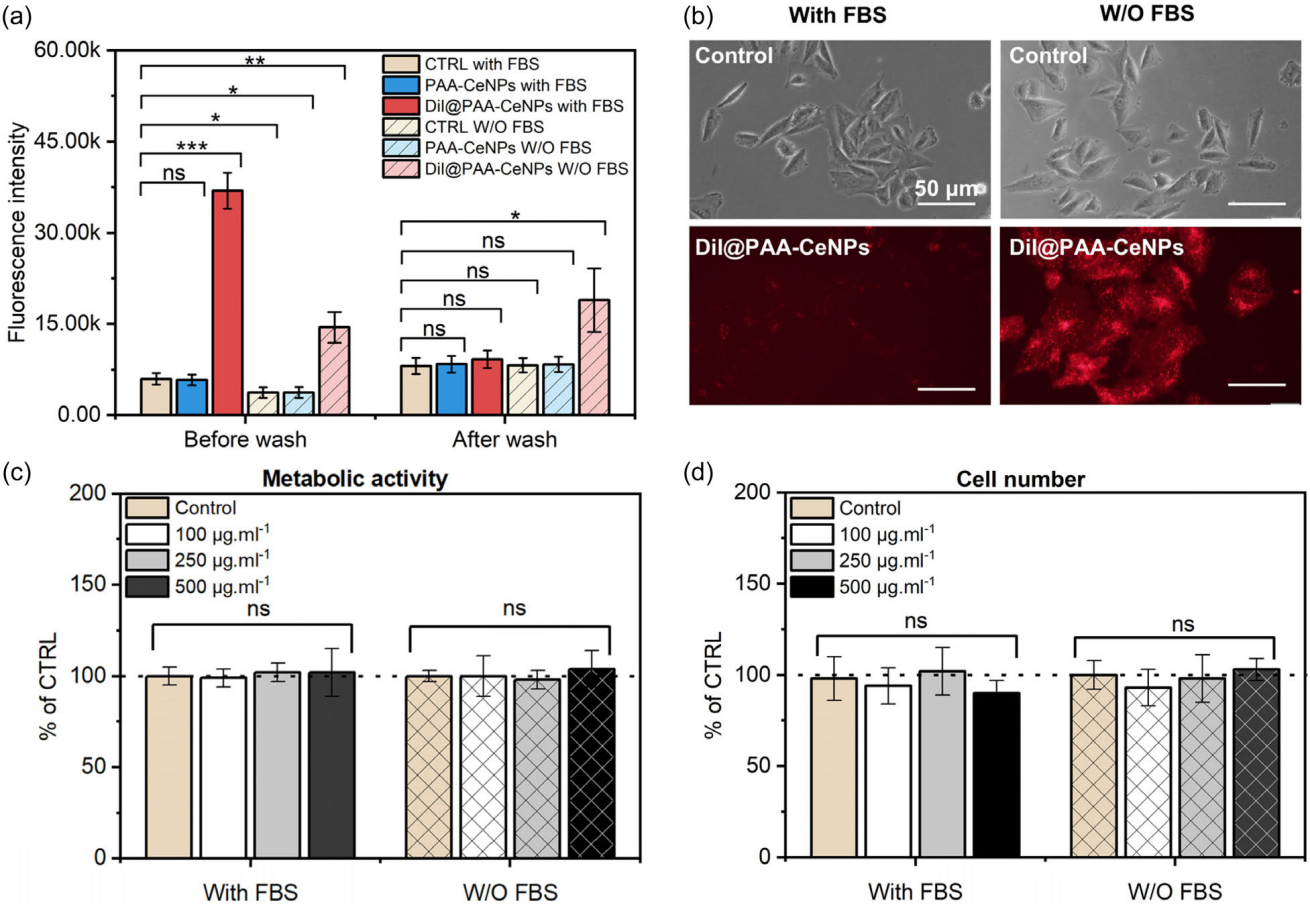
Cellular internalization and cytotoxicity assessment of PAA‐CeNPs and DiI@PAA‐CeNPs in the osteoblastic cells. a) Fluorescence intensity of different CeNPs after 1 h incubation with SAOS‐2 cells under various conditions: with or without (W/O) FBS, measured before and after washing to remove unbound NPs. b) Fluorescence microscopy images of DiI‐labeled nanoparticle (DiI@PAA‐CeNPs) coincubated with SAOS‐2 cells for 1 h with or without FBS. Scale bars = 50 μm. c) Metabolic activity of SAOS‐2 cells treated with DiI@PAA‐CeNPs for 24 h at varying NP concentrations. d) Cell number of SAOS‐2 cells treated with DiI@PAA‐CeNPs for 24 h at varying NP concentrations. Data in (c) and (d) represent mean ± standard deviation from at least three independent experiments. Statistical analysis was performed using one‐way ANOVA; no significant differences were observed (*P* > 0.05). ns represents no significant difference (*P* > 0.05), and asterisks indicate statistical significance: ^*^ *P* < 0.05, ^**^ *P* < 0.01, and ^***^ *P* < 0.001. The dashed line indicates 100% relative to the control.

Evaluation of the cytotoxicity of PAA‐CeNPs and DiI@PAA‐CeNPs after cellular internalization is essential before their application in nanomedicine.^[^ ^45^ ^]^ As an initial step, we assessed the cellular uptake of our synthesized CeNPs in the presence or absence of FBS, respectively, using the human osteosarcoma cell line SAOS‐2. As shown in **Figure** **3**a, following one hour of incubation with DiI@PAA‐CeNPs, quantitative fluorescence measurement revealed strong fluorescence in the presence of FBS. However, this fluorescence was largely lost after washing the cells, indicating that in the presence of serum proteins, the NPs were primarily adsorbed onto the cell membrane and readily removed after the washing. In contrast, under serum‐free conditions, the fluorescence signal also increased initially, but these samples retained a strong fluorescence signal even after washing, indicating effective nanoparticle internalization within 1 h. Figure 3b shows that the fluorescence signal from DiI@PAA‐CeNPs can also be observed by fluorescence microscopy at the same time (1 h). Notably, the fluorescence intensity was higher in the cells incubated in the serum‐free medium (without FBS) compared to those incubated in the presence of FBS. These results suggest that serum proteins influence cellular interactions with DiI@PAA‐CeNPs, likely by interacting with NPs in ways that hinder cellular uptake, alter membrane adsorption, and promote removal during washing. Conversely, in the absence of serum proteins, the NPs are readily internalized and retained within the cells, as confirmed by both imaging and quantitative fluorescence analysis.^[^ ^48^, ^55^, ^56^ ^]^

We further evaluated the cytotoxicity of these CeNPs using the same cell line. Cellular metabolic activity was assessed after cell coincubation with varying concentrations of DiI@PAA‐CeNPs (100, 250, and 500 μg ml^−1^) for 24 h, under conditions with or without FBS during the initial 6 h (Figure 3c). Statistical comparison shows no statistically significant difference in cytotoxicity of these NPs compared with the control. This observation was further supported by cell number assessment (Figure 3d), which also revealed no statistically significant differences between treated and control groups at any of the tested concentrations. Although FBS is known to induce nanoparticle agglomeration, potentially altering cytotoxic responses depending on nanoparticle chemistry and surface composition,^[^ ^40^, ^45^ ^]^ the tested DiI@PAA‐CeNPs did not show cytotoxicity under any conditions, similar to size‐comparable nanodiamond NPs with oxygen‐treated surface.^[^ ^57^ ^]^ In summary, these findings indicate that DiI@PAA‐CeNPs, at these concentrations, are biocompatible, and the absence/presence of FBS has no additional impact on them. Cytotoxicity of PAA‐CeNPs was already tested in our previous study, and similarly, no cytotoxic effect of the tested concentrations was detected.^[^ ^45^ ^]^ However, the presence of FBS proteins significantly affects the interaction of DiI@PAA‐CeNPs with cells. Cellular internalization of particles typically begins with the interaction at cell membranes, with particle internalization into cells being realized through different pathways—passive penetration or active endocytosis mechanisms.^[^ ^58^ ^]^ Thus, the protein presence modifies the primary nonspecific adsorption of DiI@PAA‐CeNPs on cells.^[^ ^59^ ^]^

### 2.4 Fluorescence‐Based Assessment of the Cellular Uptake Kinetics of DiI@PAA‐CeNPs

**Figure 4:**
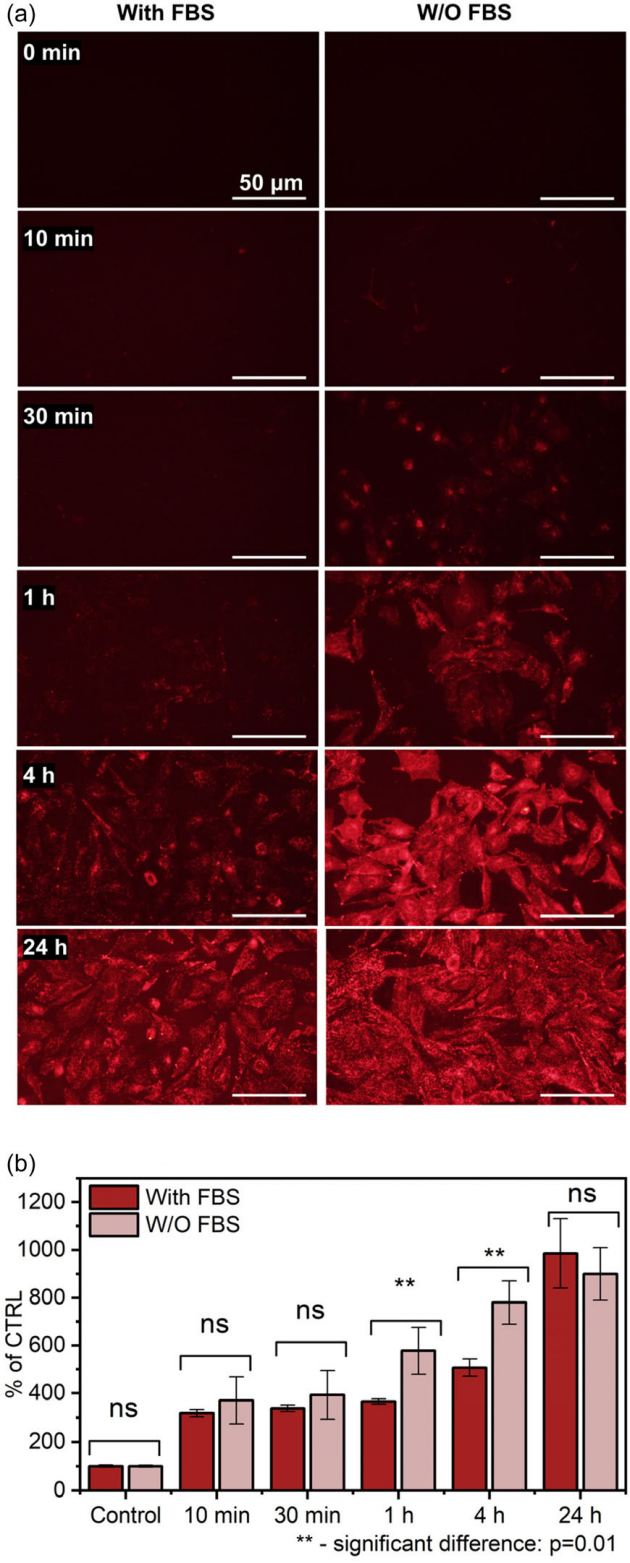
Time‐dependent cellular uptake kinetics of DiI@PAA‐CeNPs in the osteoblastic cells. a) Fluorescence microscopy images showing cellular uptake of DiI@PAA‐CeNPs over time (0 min, 10 min, 30 min, 1 h, 4 h, and 24 h), with or without FBS. Scale bars represent 50 μm. b) Quantitative analysis of DiI@PAA‐CeNPs uptake kinetics measured by spectrofluorometry. Asterisks indicate statistically significant differences between cells incubated with or without FBS (^**^ *p* = 0.01). Data represents mean value ± standard deviation from at least three independent experiments. Statistical analysis was performed using one‐way ANOVA; ns represents no significant difference (*P* > 0.05), and asterisks indicate statistical significance: ^**^ *P* < 0.01.

Since there was a significant difference in fluorescence signal of DiI@PAA‐CeNPs after 1 h incubation (Figure 3b) in the cells cultivated in the medium with or without FBS, further cellular uptake kinetics experiments with these fluorescence NPs were performed. DiI@PAA‐CeNPs (100 μg ml^−1^) were incubated with osteoblastic cells under different conditions and for different times. Quantification of nanoparticle internalization by the cells was performed using fluorescence microscopy (**Figure** **4**a) as well as the spectrofluorometer (Figure 4b). After 1 h of incubation with DiI@PAA‐CeNPs, strong fluorescence was observed in the cells cultivated without FBS, indicating rapid NP uptake. In contrast, the cells incubated in the presence of FBS showed delayed and significantly weaker fluorescence, suggesting reduced or slower internalization. At both 1 and 4 h, the fluorescence intensity in the without‐FBS group was significantly higher than in the group with FBS (*p* = 0.01), confirming greater NP uptake in the absence of FBS. However, within 24 h, fluorescence levels under both conditions converged, indicating that over time, similar levels of nanoparticle internalization were eventually achieved regardless of FBS presence. Collectively, our results reveal a significant link between protein presence and NP internalization^[^ ^48^, ^56^ ^]^ while NP uptake increases overall with time, the presence of FBS modulates the rate of internalization during the early phases. Specifically, the presence of FBS transiently slows the initial uptake of DiI@PAA‐CeNPs; however, with prolonged incubation, nanoparticle internalization reaches similar levels under both serum‐containing and serum‐free conditions. The initial delay in uptake under serum‐containing conditions may reflect protein corona effects, as suggested in previous studies, including our work on nanodiamond NPs.^[^ ^57^ ^]^ However, the corona on DiI‐labeled CeNPs has not been directly characterized, and a more detailed mechanistic study would require advanced single‐protein analysis and is beyond the scope of the present work. The convergence of uptake at 24 h likely results from complete internalization of the added NPs, as both DiI@PAA‐CeNPs and PAA‐CeNPs were added at the same concentration and the confluent cell layer limits further accumulation.

### 2.5 Intracellular Distribution of DiI@PAA‐CeNPs Using Fluorescence Microscopy

**Figure 5:**
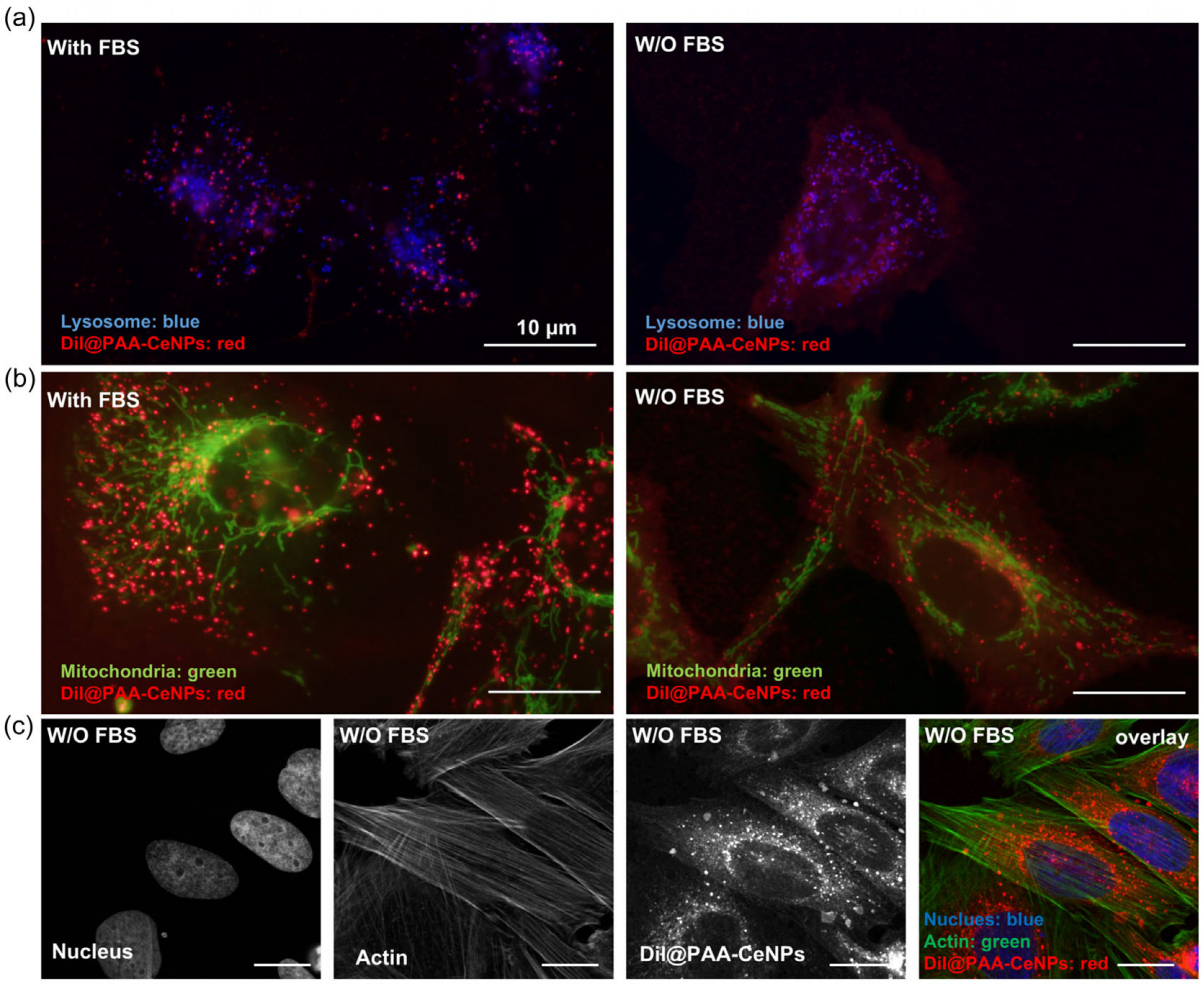
Intracellular localization of DiI@PAA‐CeNPs in SAOS‐2 cells. Fluorescence microscopy images of fluorescently stained a) lysosomes (blue) and b) mitochondria (green) after the cell incubation with DiI@PAA‐CeNPs (red) in the medium with or without FBS for 4 h. c) Confocal laser scanning microscopy images of the cells incubated with DiI@PAA‐CeNPs (red) for 24 h (initial 6 h without FBS supplementation), stained for nuclei (DAPI: blue) and actin (phalloidin: green). Scale bars are 10 μm.

After 4 h of DiI@PAA‐CeNPs incubation with the cells, the NPs were detected inside the cells under both conditions (Figure 4b) but provided different intensity of fluorescence signal (more intensive without FBS). A more thorough analysis of their distribution was carried out. **Figure** **5**a shows that the overall fluorescence signal differs depending on the type of incubation after 4 h. In the cells incubated with FBS (Figure 5a, left), the NPs formed distinct red punctate structures that were distributed throughout the cell, suggesting their accumulation within specific vesicles. However, these red signals from DiI@PAA‐CeNPs are distributed regardless of fluorescently stained lysosomes (blue). These were primarily localized around the nucleus. Thus, we can speculate that no colocalization between DiI@PAA‐CeNPs and lysosomes took place after 4 h of incubation in the presence of FBS. However, the NPs were localized in some vesicles, which can be early or late endosomes, because the trafficking of NPs was significantly slowed down.^[^ ^60^ ^]^ In contrast, the cells cultured without FBS (Figure 5a, right) showed a different distribution of the DiI@PAA‐CeNPs. The fluorescence signal appeared mostly scattered or diffused with occasional puncta, some of which seemed to overlap with stained lysosomes. These observations suggest that DiI@PAA‐CeNPs in the absence of FBS in the medium underwent a distinct intracellular entry and trafficking pathway compared to those incubated with FBS proteins. In the absence of FBS, the diffused cytoplasmic fluorescence implies potential direct membrane penetration to the cytosol of a subset of NPs, as already shown for 2 nm SiQDs incubated with or without FBS.^[^ ^61^ ^]^ However, the presence of occasional punctate signals, in some cases overlapping with lysosomes, also indicates that a subset of these NPs was internalized via the endosomal/lysosomal pathway, and some of them finished already in lysosomes. Overall, these images demonstrate that FBS proteins may hinder/slow down the internalization and endo/lysosomal trafficking of DiI@PAA‐CeNPs, whereas serum‐free conditions may facilitate more rapid and extensive nanoparticle accumulation within cells, including their dispersed distribution in cytoplasm as well as localization to endo/lysosomal vesicles already by 4 h.

As shown in Figure 5b, the cells incubated with DiI@PAA‐CeNPs for 4 h and stained for mitochondrial distribution show clear spatial separation between the nanoparticle‐derived red fluorescence and the green mitochondrial network, indicating no apparent colocalization. The reticular, filamentous pattern typical of active mitochondria was dispersed throughout the cytoplasm, while the DiI‐positive signal is localized in distinct puncta in the case of FBS presence, but it is mostly diffused with few discrete puncta in the absence of FBS, as presented previously. These findings suggest that DiI@PAA‐CeNPs do not localize to, or disrupt, mitochondria at early stages of their internalization and are instead restricted to non‐mitochondrial, endosomal/lysosomal compartments or are freely in the cytoplasm.

Figure 5c shows the intracellular localization of DiI@PAA‐CeNPs after 24 h of coincubation with SAOS‐2 cells, as assessed by confocal laser scanning microscopy. Efficient DiI@PAA‐CeNPs internalization was observed without noticeable disruption of nuclear morphology or cytoskeletal integrity. Nuclei appear uniformly stained with DAPI and retain their characteristic elliptical shape with no evidence of micronuclei or chromatin condensation, indicating that DiI@PAA‐CeNPs exposure under these conditions does not induce detectable genotoxic stress.^[^ ^62^ ^]^ Phalloidin labeling of F‐actin reveals an intact, well‐organized actin cytoskeleton characterized by a dense fiber network; there is no observable disruption or fragmentation of actin filaments, suggesting that cellular contractility and overall morphology remain uncompromised. The DiI@PAA‐CeNPs signal appears as numerous, small, punctate foci dispersed throughout the cytoplasm, with a tendency to accumulate in the perinuclear region, suggesting their localization to the lysosomes. While brief observations suggest possible lysosomal localization of DiI‐labeled CeNPs, detailed colocalization studies with endo/lysosomal markers were not performed, as precise subcellular localization was beyond the scope of this study. Importantly, these red fluorescent puncta do not colocalize with the nuclear DAPI signal, confirming that the NPs remain excluded from the nucleus. These findings further indicate that DiI@PAA‐CeNPs are efficiently internalized by SAOS‐2 cells, localize to perinuclear compartments, and exhibit sustained intracellular retention without compromising mitochondrial function, cytoskeletal architecture, or nuclear integrity.

In conclusion, fluorescence analysis reveals that the presence of FBS proteins altered both the kinetics and intracellular localization of DiI@PAA‐CeNPs. Specifically, the presence of FBS in the cultivation medium with DiI@PAA‐CeNPs delayed the onset of nanoparticle uptake (kept them on the surface for a longer time) and internalized them via endocytosis in vesicles, whereas in FBS absence, NPs were internalized more rapidly and can be found dispersed in the cytoplasm as well as localized within lysosomes.

### 2.6 Observing NPs Intracellular Distribution via TEM

One critical assumption when employing fluorescence microscopy to visualize dye‐labeled nanozymes within cells is that the fluorescent tag remains stably adsorbed onto the nanoparticle and does not undergo detachment, degradation, or independent redistribution, which could otherwise lead to misinterpretation of nanoparticle localization. Our dialysis experiments (Figure S1, Supporting Information) confirmed that DiI remained stably associated with DiI@PAA‐CeNPs for over 24 h in solution, with no detectable dye release. While Figure S1, Supporting Information indicates minimal dye leakage in solution, intracellular conditions may alter dye stability. We therefore acknowledge that the potential for intracellular dye dissociation could affect fluorescence‐based observations and interpretations. Would the DiI@PAA‐CeNPs locate differently than PAA‐CeNPs because of the presence of DiI? At present, alternative visualization techniques capable of distinguishing between dyed and nondyed NPs within cells remain limited.

To complement the fluorescence‐based assessment of cellular uptake, we further quantified the internalized cerium content using inductively coupled plasma mass spectrometry (ICP‐MS). After 24 h incubation with PAA‐CeNPs and DiI@PAA‐CeNPs, cell lysates were collected and analyzed for Ce concentration. The measured Ce levels were 1.46 ± 0.36 μg ml^−1^ for PAA‐CeNPs and 1.97 ± 0.46 μg ml^−1^ for DiI@PAA‐CeNPs, showing no statistically significant difference between the two groups. These comparable values indicate that the presence of DiI labeling does not significantly alter the amount of nanoparticle internalization after 24 h. The ICP‐MS results thus provide a label‐free, quantitative benchmark that validates fluorescence observations and supports a consistent uptake behavior across both formulations.

**Figure 6:**
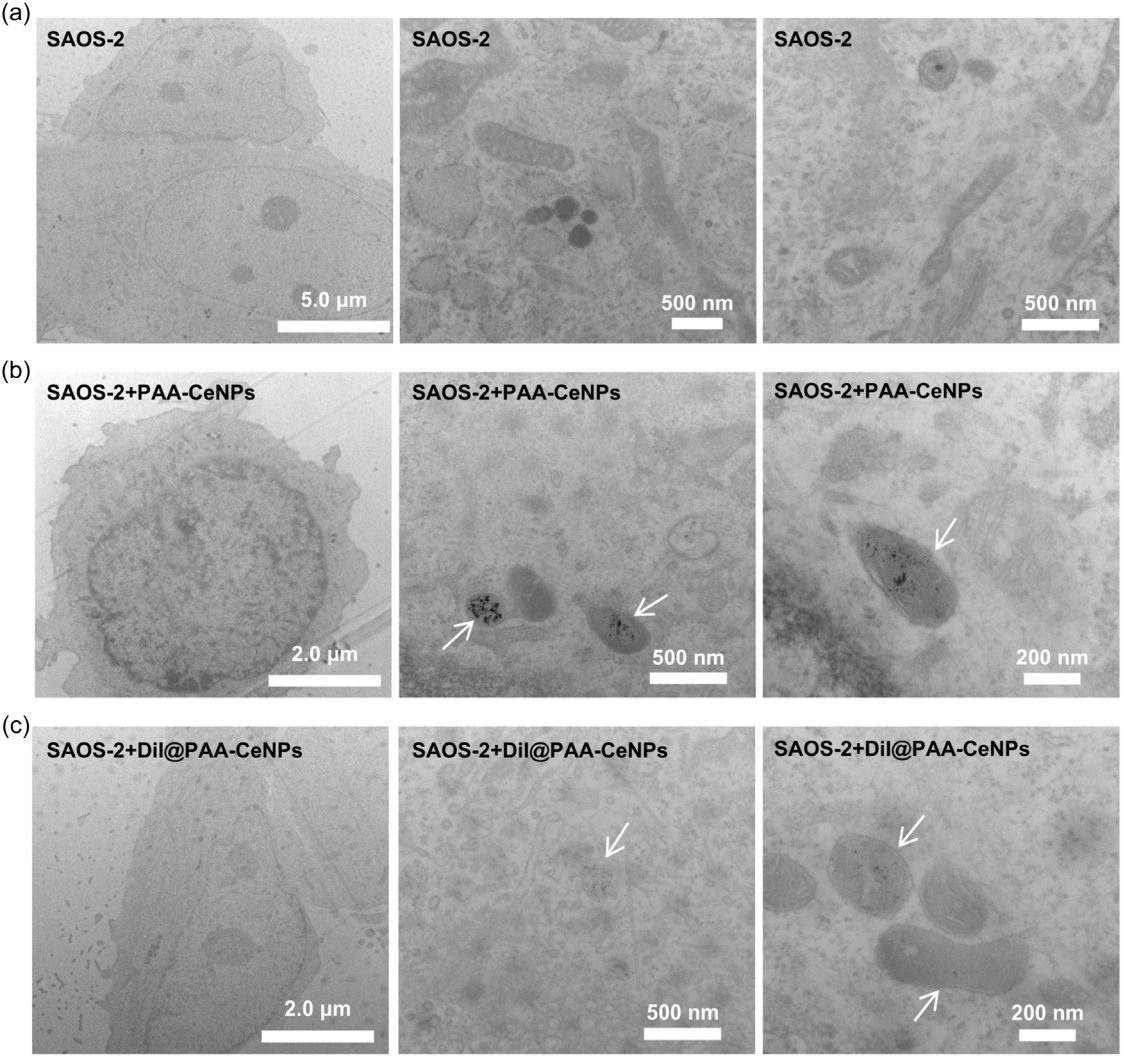
Representative TEM images of SAOS‐2 cells coincubated with CeNPs. a) Control SAOS‐2 cells, showing typical cellular structures and organelles. b) SAOS‐2 cells coincubated with PAA‐CeNPs. c) SAOS‐2 cells coincubated with DiI@PAA‐CeNPs. Under all conditions, the cells were supplemented with FBS and incubated with NPs for 4 h. Arrows indicate observed NPs within cellular vesicles.

Since nondyed particles are not visible using fluorescence‐based microscopy, traditional TEM combined with negative staining remains the most widely used method for visualizing NPs inside cells. We investigated the NPs’ internalization inside the cells via TEM (**Figure** **6**) to provide a complementary comparison to those observed via fluorescence‐based microscopy. SAOS‐2 cells incubated for 4 h with either PAA‐CeNPs or DiI@PAA‐CeNPs in the presence of FBS exhibited clear evidence of both nanoparticle uptake and intracellular localization. Importantly, no ultrastructural abnormalities were observed in the cells, further confirming that the NPs were well tolerated under the tested conditions. In the untreated control cells (Figure 6a), the cytoplasm appeared homogeneous, with well‐preserved ultrastructure and clearly defined organelles, including mitochondria, endoplasmic reticulum, and vesicular compartments. The nuclear envelope remained intact, indicating that the cells maintained normal morphology under basal conditions. For the cells incubated with PAA‐CeNPs, TEM reveals the presence of aggregated CeNPs predominantly localized within membrane‐bound intracellular vesicles (Figure 6b). These vesicles displayed relatively uniform internal ultrastructure and increased electron density, consistent with the accumulation of nanoparticle aggregates. Importantly, surrounding organelles exhibited normal morphology: mitochondrial cristae appeared well‐organized, and no significant vacuolization, membrane rupture, or other ultrastructural abnormalities were noted. For cells incubated with DiI@PAA‐CeNPs (Figure 6c), intracellular electron‐dense deposits of comparable size and vesicular distribution were observed. However, compared with PAA‐CeNPs, the cells incubated with DiI@PAA‐CeNPs appeared to contain fewer visible particle aggregates.

To conclude, the electron‐dense PAA‐CeNPs and DiI@PAA‐CeNPs both appeared to be localized within specific but unidentified organelles with relatively uniform inner ultrastructure and membrane. The markedly lower fluorescent intensity of DiI@PAA‐CeNPs observed inside cells compared to PAA‐CeNPs may suggest that DiI loading may reduce the overall cellular uptake or alter NP trafficking and retention. This could be attributed to changes in their surface properties, stability, or interaction with cellular membranes caused by the DiI incorporation. Notably, freely dispersed NPs were not detected in the cytosol in any case. Negative staining with heavy metals like osmium (Os) primarily enhances the contrast of biological structures such as membranes and proteins but can sometimes obscure or reduce the contrast of individual NPs, especially if they are very small or not aggregated.^[^ ^63^ ^]^ This makes detecting single, well‐dispersed nanozymes challenging at typical TEM resolutions under negative staining conditions. While fluorescence microscopy indicates rapid uptake of DiI@PAA‐CeNPs, particularly in serum‐free conditions, TEM confirms their internalization but may undercount small or dispersed particles. Similarly, fluorescence intensity reflects overall labeling rather than absolute particle number, and these limitations should be considered when interpreting cellular uptake data.

### 2.7 High‐Resolution Vertical Cross‐Section Imaging of Dyed and Nondyed CeNPs in the Cells

Dual‐beam focused ion beam and scanning electron microscopy (FIB‐SEM) assisted lamella preparation for scanning TEM (STEM) observation enables vertical cross‐sectioning of the cells incubated with the NPs, allowing analysis of NPs distribution throughout the entire cell thickness, from one membrane surface, through the cytoplasm and nucleus, to the opposite membrane.^[^ ^64^, ^65^ ^]^ Unlike traditional TEM and STEM, which typically involve horizontal ultrathin sectioning and often require negative staining (potentially obscuring or altering NP visibility), FIB‐SEM lamella for STEM observation provides high‐resolution imaging to distinguish electron‐dense NPs without the need for staining.

**Figure 7:**
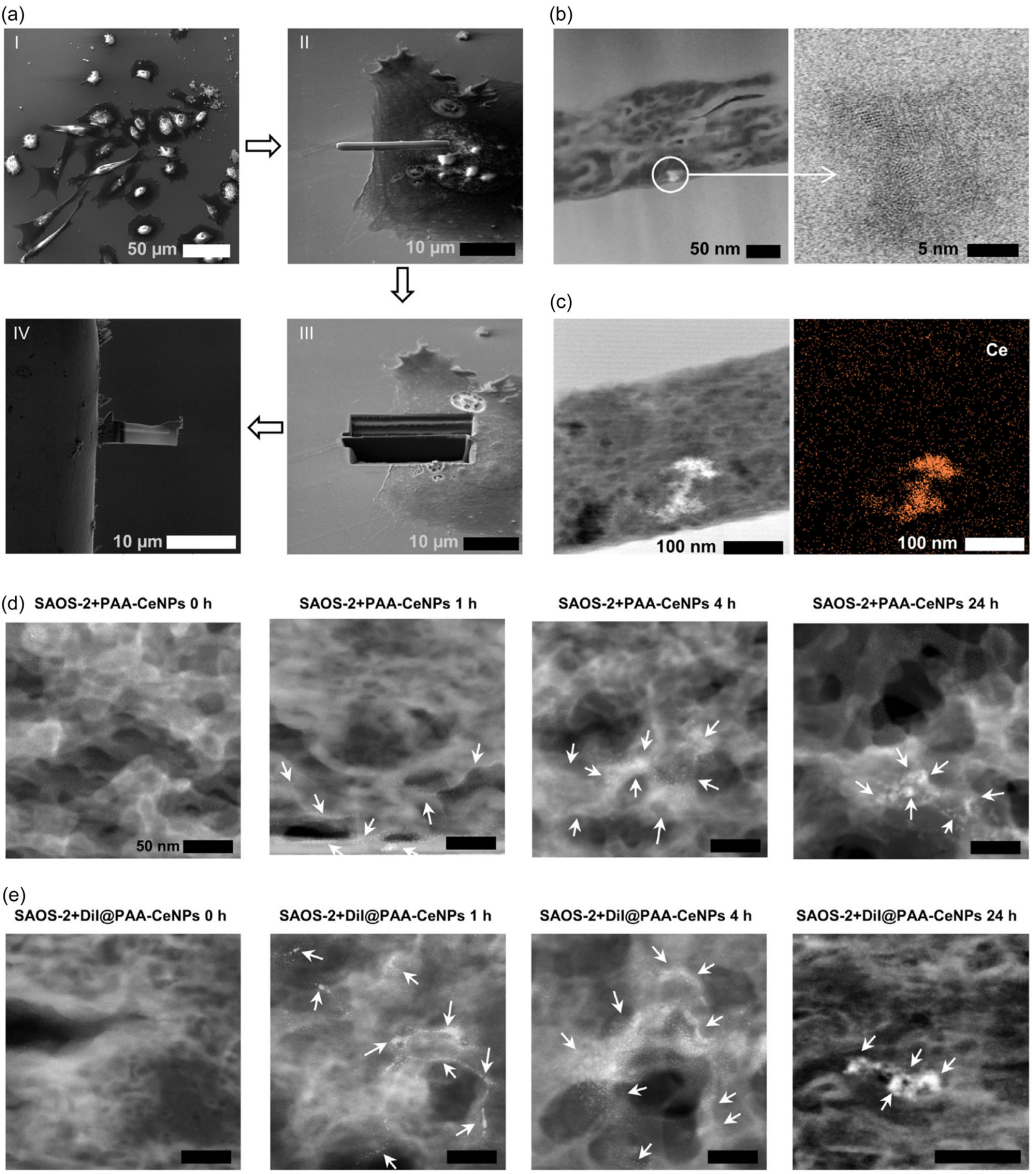
Focused ion beam (FIB)‐assisted lamella preparation and visualization of internalized PAA‐CeNPs and DiI@PAA‐CeNPs via HR‐STEM. a) Workflow of FIB‐SEM preparation for thinning fixed cells to produce lamella slices: (I) SEM localization of target cells; (II) deposition of a protective layer before milling; (III) focused Ga ion beam milling of slices from fixed cells; (IV) mounting of thinned lamellae onto TEM holders for analysis followed by focused Ga ion beam polishing until the desired thickness is achieved. b) Observation of CeNPs inside the cells by HR‐STEM and HR‐TEM. The detailed crystalline structure of CeNPs is visible. c) STEM images (left) and EDX mapping (right) confirm the existence of Ce inside the sliced cell lamella. Cells were incubated with NPs for 24 h in the medium supplemented with FBS. d,e) shows the intracellular localization of CeNPs in SAOS cells at various time points, analyzed using HR‐STEM following FIB‐SEM‐assisted lamella preparation. (d) PAA‐CeNPs and (e) DiI@PAA‐CeNPs incubated with SAOS‐2 cells in FBS‐supplemented cultivation medium for 0 to 24 h. White arrows indicate observed bright‐contrasted CeNPs observed in the FIB‐SEM prepared lamellae. Size of scale bars: 10 μm, 50 μm, 5 nm, 50 nm, and 100 nm, as indicated. For (d) and (e), scale bars are 50 μm unless otherwise indicated.

We have subsequently applied FIB‐SEM to prepare cell lamellae in the vertical direction. Before lamellae preparation, the morphology of cells incubated with PAA‐CeNPs and DiI@PAA‐CeNPs was checked by growing them under the same conditions. As shown in Figure S2, Supporting Information, the presence of NPs did not induce any observable morphological alterations in the cells. **Figure** **7**a illustrates the in situ FIB‐SEM workflow for preparing a lamella from the cells cultured with the NPs. A target cell is first located and imaged by scanning electron microscope (SEM), then a thin protective layer of silicon oxide is deposited over the region of interest to prevent ion damage. Using a high‐voltage focused Ga beam, trenches were milled on either side of this protected area to isolate a slab of material while preserving the ultrastructure of interest. Finally, the lamella was welded to the nanomanipulator, cut free from the bulk, transferred onto a TEM lift‐out grid, and polished from both sides until achieving the desired thickness. This procedure yielded an electron‐transparent lamella containing the cell of interest for high‐resolution STEM (HR‐STEM) analysis. A HR‐STEM image of a cross‐section through a SAOS‐2 cell shows visibly internalized PAA‐CeNPs in Figure 7b. The left micrograph (low‐magnification) highlights a particulate inclusion (circled), and the inset (high‐magnification) on the right shows well‐resolved crystal lattice fringes within CeNPs. These lattice spacings correspond to the fluorite crystal structure of cerium oxide; for example, the dominant (111) plane fringes are visible. The presence of sharp lattice fringes confirms that the internalized CeNPs remained crystalline after their uptake by the cell. In Figure 7c, energy‐dispersive X‐ray spectroscopy (EDX) mapping of a bright cluster reveals the presence of cerium, indicating the localization of CeNPs within the cellular environment. While distinct vesicular structures were not identifiable, the elemental map supports the presence of Ce‐containing particles inside the cell. Figure 7d,e show time‐course series of HR‐STEM images of FIB lamellae illustrating the interaction between PAA‐CeNPs and DiI@PAA‐CeNPs with SAOS‐2 cells at 0‐, 1‐, 4‐, and 24‐h of incubation. When using a dark‐field detector in HR‐STEM, the electron‐dense CeNPs appeared as bright regions due to enhanced scattering of electrons.

At the beginning, no intracellular CeNPs were observed, serving as a negative control. Both PAA‐CeNPs and DiI@PAA‐CeNPs show increased cellular uptake over time, as evidenced by the accumulation and internal distribution of bright, electron‐dense signals within the cells. After 1 h, in both groups, most NPs appeared freely distributed within intracellular regions resembling the cytosol, with a few clusters observed, suggesting the onset of cellular internalization. At 4 h, more freely distributed NPs as well as clusters were observed in both groups, although neither formed large aggregates. By 24 h, both CeNPs appeared as discrete electron‐dense spots, with a higher degree of aggregation than that of 4 h.

Fluorescence microscopy, widely used to visualize nanoparticle localization, primarily detects only aggregates due to its limited resolution, as shown in the case of FBS presence. Similar limitations—artifacts and misinterpretation of nanoparticle localization—have been reported in conventional fluorescence imaging, which is bound by the diffraction limit of ≈200 nm, making it difficult to resolve particles below this threshold.^[^ ^66^ ^]^ Negatively stained samples observed by traditional TEM show more PAA‐CeNPs within vesicles compared to DiI@PAA‐CeNPs. While negative staining‐based TEM preserves overall cellular structure and provides an overview of NP compartmentalization, it cannot detect individual NPs satisfactorily due to limited contrast. Negative staining suffers from limited contrast and resolution due to the granular nature and uneven distribution of heavy‐metal stains, which hampers the reliable detection of individual NPs, particularly those under ≈10 nm.^[^ ^67^ ^]^ Moreover, cellular structures and stain precipitates can mimic nanoparticle morphology, increasing the risk of misidentification without elemental confirmation. In contrast, HR‐STEM characterization of FIB‐prepared lamellae reveals individual and small clusters of CeNPs in the cytosol. FIB–prepared lamellae, followed by HR‐STEM imaging, allow direct visualization of individual or small clusters of NPs within the cellular matrix, although using such a combination is not commonly reported due to the tedious workload and preparation procedures. For instance, FIB/SEM combined with STEM enables nanometer‐resolution imaging of the cell–material interface, capturing details like membrane deformation, organelles, and nanoscale interactions with synthetic materials.^[^ ^68^ ^]^ Similarly, cryoFIB–SEM has captured internalized gold–conjugated labels in endo–lysosomal vesicles of HeLa cells using back–scattered electron contrast.^[^ ^69^ ^]^ Here we note that although HR‐STEM in combination with FIB‐prepared lamellae provided high‐resolution but only localized 2D views. Additionally, correlative FIB‐SEM combined with STEM tomography can enable nanoscale 3D reconstruction of resin‐embedded cells as reported, allowing visualization of intracellular structures—including NPs—within thicker lamellae.^[^ ^70^ ^]^ Nevertheless, combining fluorescence microscopy, negative staining‐based TEM, and FIB lamellae‐based HR‐STEM delivers a comprehensive picture of nanoparticle distribution,^[^ ^21^ ^]^ capturing both aggregation and free dispersion. A summary of the advantages and limitations of these imaging techniques is provided in **Table** **1**.

**Table 1:** Comparison of methods used for nanoparticle visualization within cells.

Technique	Pros	Cons
Fluorescence microscopy	‐ Live cell imaging ‐ High throughput ‐ Multiplex labeling	‐ Needs dye ‐ Low resolution ‐ Cannot detect unlabeled NPs ‐ Lowest resolution, difficult to detect individual NPs
Negative staining + TEM	‐ High resolution ‐ Good vesicle/membrane contrast	‐ Difficult to detect individual NPs ‐ Horizontal sections mostly ‐ Staining artifacts
FIB‐SEM lamellae + STEM	‐ Vertical sectioning view ‐ Detects unlabeled NPs ‐ Sub‐nm resolution ‐ No stain needed	‐ Harsh preparation ‐ Low throughput ‐ Poor vesicle/organelle context ‐ Lacks cellular structure overview

## 3 Conclusion

This study provides critical insights into the often‐overlooked question, “to dye or not to dye?”—demonstrating that fluorescent labeling, while essential for nanoparticle and nanozyme tracking, is not biologically neutral. Such labeling‐induced alterations are consistent with prior observations that surface functionalization or protein corona formation^[^ ^21^, ^22^ ^]^ can modulate nanozyme catalytic activity and uptake behavior by altering substrate accessibility and particle–cell interactions. However, to the best of our knowledge, no previous studies have directly examined the influence of fluorescent tags on these processes as presented in this study.

In this work we investigated a specific system, combining a single nanomaterial (CeNPs) and a single dye (DiI), and investigated the impact of such fluorescence tag on the intracellular behavior of these CeNPs. Although TEM shows a modest difference in core size between PAA‐CeNPs (2.4 nm) and DiI‐labeled PAA‐CeNPs (3.7 nm), the hydrodynamic diameters in biological media, particularly in DMEM supplemented with FBS, are comparable (≈25 nm), reflecting the effective size experienced by cells. Functionalization of poly(acrylic acid)‐coated cerium oxide nanozymes with the lipophilic dye DiI preserved most of their redox activity and biocompatibility but altered cerium surface oxidation states and cellular uptake kinetics, likely due to interaction with serum proteins, although the influence on their final cellular fate is not significant.

Fluorescence microscopy revealed that the presence of serum proteins delays the uptake of DiI@PAA‐CeNPs, leading to their prolonged surface retention and internalization. In serum‐free conditions, DiI‐labeled CeNPs were internalized more rapidly and appeared both in the cytoplasm and within endosomes/lysosomes. TEM with negative staining observation confirmed vesicular localization of both PAA‐CeNPs and DiI@PAA‐CeNPs but failed to detect cytosolic particles, likely due to staining‐related contrast limitations. In contrast, HR‐STEM of FIB‐SEM‐prepared lamellae enabled clear detection of freely dispersed CeNPs across the vertical cell distribution. At early time points, cytosolic localization was evident. Over time, both NP types showed increased clustering. By 24 h, electron‐dense NP clusters were prominent in both groups with no significant differences, in agreement with the ICP‐MS results indicating no significant difference in the amount of nanoparticle uptake. Our study highlights the importance of carefully selecting complementary characterization techniques, such as fluorescence‐based methods and/or TEM, as well as sample preparation for electron microscopes (native staining of sectioned cells or FIB‐assisted lamellae preparation), since each approach has distinct advantages and limitations that must be considered to accurately interpret NP behavior in cells.

Integrating label‐free, high‐resolution imaging with fluorescence techniques is crucial for accurately characterizing nanozyme–cell interactions. In this work, we focused on PAA‐coated cerium oxide NPs labeled with a single dye, which may have limited generalizability to all NPs systems. However, we show that fluorescent labeling can introduce significant artifacts to CeNPs, highlighting the need for careful physicochemical and functional evaluation of labeled nanomaterials. Beyond this specific system, the imaging and analysis workflow combining advanced fluorescence microscopy, TEM, FIB‐SEM lamella preparation, and HR‐STEM is broadly transferable to other nanozyme or nanomaterial studies. The decision to dye any NPs must balance imaging convenience against potential alterations in nanozyme behavior within complex biological environments, a critical consideration for the rational development of multifunctional nanozymes in diagnostic and therapeutic applications.

## 4 Experimental Section

4.1

4.1.1

##### Synthesis of PAA‐CeNPs and DiI@PAA‐CeNPs

Unless otherwise specified, all chemicals were obtained from Sigma‐Aldrich (Merck). PAA‐CeNPs were synthesized via a wet‐chemical precipitation method at 25 °C, following previously established protocols.^[^ ^40^, ^45^ ^]^ For DiI@PAA‐CeNPs, 10 μl of 1 mg ml^−1^ of DiI (in dimethyl sulfoxide, Thermo Fisher Scientific) was added to 1 mL of PAA‐CeNPs at the same concentration. The NPs were further dialyzed with a 10 kDa MWCO membrane (Thermo Fisher Scientific) overnight. The dialysates at 0 h and 24 h were collected and analyzed by a Multiskan Go UV–visible (UV–Vis) spectrometer (Thermo Fisher Scientific) to detect dye leakage. Concentrations of both DiI and CeNPs were calibrated UV–Vis spectrometer, where the absorptivity coefficient of cerium oxide at 288 nm was around 25.2 L g^−1^ cm^−1^, and DiI at 556 nm was around 76 L g^−1^ cm^−1^. DiI was incorporated into PAA‐coated cerium oxide NPs via physical encapsulation, relying on hydrophobic embedding within the polymer coating, without covalent bonding.

##### Characterization of PAA‐CeNPs and DiI@PAA‐CeNPs

The optical and physicochemical properties of PAA‐CeNPs and DiI@PAA‐CeNPs were characterized using multiple analytical techniques. UV–Vis absorption spectra were recorded with a Multiskan GO UV–Vis spectrometer with a microplate reader (Thermo Fisher Scientific) with absorbance from 200 nm to 800 nm at 1 nm per step. XPS was used to analyze the surface chemistry and oxidation states of CeNPs. NP dispersions were air‐dried at 50 °C for 72 h, ground into fine powders, and pressed onto 0.2 mm‐thick high‐purity indium foil (99.99%, Lesker) for analysis. The instrument was equipped with a monochromatized Al *Kα* X‐ray source (1486.6 eV) and a multichannel electron energy analyzer (Specs Phoibos 150) with a differentially pumped electrostatic prelens system. Core‐level spectra for Ce 3*d*, C 1*s*, and O 1*s* were acquired. Spectral deconvolution and fitting were performed using KolXPD software. XRD diffractograms were collected using a Bruker DX Advance diffractometer equipped with a Vantec‐1 detector in the Bragg‐Brentano geometry using Cu *Kα* radiation. DLS and zeta potential analysis were conducted using a Zetasizer Pro (Malvern Instruments). The hydrodynamic diameter was reported as number‐weighted averages. High‐resolution scanning TEM (STEM) imaging was performed using JEOL JEM‐2200FS and an aberration‐corrected JEOL NEOARM 200F microscopes with a Schottky‐type field emission gun at an accelerating voltage of 200 kV using a high‐angle annular dark‐field (HAADF) detector. Both the CeNPs samples and the cell lamella samples were characterized by STEM. Corresponding EDX maps were acquired using a JEOL JED‐2300 detector. For NP samples, diluted CeNPs solutions were drop‐cast onto a holey carbon‐coated copper 300‐mesh grid (Agar Scientific). The cell lamella sample preparation is described in the following sections. To evaluate the colloidal stability of CeNPs, their *D*_H_ and surface zeta potentials were monitored by DLS. Briefly, 1 mg ml^−1^ PAA‐CeNPs or DiI@PAA‐CeNPs (or equivalent DiI) suspension was mixed with water, original DMEM cell culture media, or DMEM supplemented with 5% FBS. The measurement of DLS with DMEM (with or without FBS) was performed using 1 mL DMEM media (with or without 5% FBS) mixed with 100 μl of 1 mg ml^−1^ NPs aqueous solution. Detailed conditions for DLS measurements were listed in Table S1, Supporting Information. Composition of DMEM media is listed at Table S2, Supporting Information. The mixtures were vortexed thoroughly to ensure homogeneous dispersion and incubated at room temperature to allow protein–nanoparticle interactions. Following incubation, the samples were characterized by their hydrodynamic diameter and/or zeta potentials.

##### Antioxidant Activity Measurement

To assess the enzyme‐mimetic properties of synthesized NPs, their superoxide dismutase, catalase, oxidase, and peroxidase‐like activities were systematically evaluated using established colorimetric assays and commercialized kits. SOD and catalase activities were quantified using commercial enzyme assay kits (SOD Assay Kit, Cat. No. 19160; Catalase Assay Kit, Cat. No. CAT100, Sigma–Aldrich), following the manufacturer's protocols. For the SOD assay, samples were diluted to concentrations that ensured the measured enzyme inhibition rate fell within the linear dynamic range of 20%–80%. Negative controls were included to account for potential assay interference from the tested media. The assay is based on the generation of a formazan dye, whose concentration is inversely proportional to SOD activity. Absorbance at 440 nm was recorded after a 20 min reaction using a Multiskan GO UV–Vis spectrometer (Thermo Fisher Scientific). The catalase activity assay relied on the reaction of hydrogen peroxide with a phenol‐based substrate system consisting of 3,5‐dichloro‐2‐hydroxybenzenesulfonic acid and 4‐aminoantipyrine, catalyzed by horseradish peroxidase (HRP). This reaction yields a red quinoneimine dye, which was measured at 520 nm. Fresh standard curves for hydrogen peroxide were prepared for each experimental set. The oxidase‐like catalytic activity of PAA‐CeNPs and DiI@PAA‐CeNPs was evaluated using 3,3′,5,5′‐tetramethylbenzidine (TMB) as a substrate. A 10 mg ml^−1^ TMB stock solution in DMSO was diluted 100‐fold with citrate/acetate buffer to prepare the working solution. To this, 10 μl of 1 mg ml^−1^ CeNPs samples were added to 200 μl of the TMB reaction solution and incubated in the dark for 30 min. The reaction was terminated by the addition of 100 μl of 0.1 M sulfuric acid (H_2_SO_4_), resulting in a color shift from blue to yellow. Absorbance at 450 nm was used to quantify oxidase activity. Peroxidase‐like activity was assessed through a similar TMB oxidation assay, with the inclusion of hydrogen peroxide to initiate the reaction. Specifically, 4 μl of 30% H_2_O_2_ was added to the TMB reaction mixture before NP addition. Absorbance at 450 nm with and without H_2_O_2_ was used to distinguish peroxidase activity from intrinsic oxidase activity.

##### Cell Cultivation

Human osteoblastic cells SAOS‐2 (contaminationfree) were purchased on 13.9.2024 from German Collection of Microorganisms and Cell Cultures GmbH (DSMZ), RRID: CVCL_0548. The cells were maintained under standard culture conditions in McCoy's 5A medium (GE Healthcare‐HyClone), supplemented with 15% fetal bovine serum (FBS; Biosera), L‐glutamine (Life Technologies), 10 000 U·ml^−1^ penicillin, and 10 mg ml^−1^ streptomycin. The cells were incubated in a humidified incubator at 37 °C with 5% CO_2_.

##### Cellular Uptake Kinetics of DiI@PAA‐CeNPs

SAOS‐2 cells were seeded in 96‐well plates at a density of 20 000 cells cm^−2^ and allowed to adhere for 24 h in the standard growth medium. Following this preincubation, the cells were gently washed with phosphate‐buffered saline (PBS; Thermo Fisher Scientific‐Gibco), and a fresh medium containing DiI@PAA‐CeNPs (with 5% FBS or without FBS) was applied at different time intervals to finish the experiment at the same time (minus 4 h, 1 h, 30 min, 10 min and control at the time of measurement) on one plate. On a separate 96‐well plate, the cells with DiI@PAA‐CeNPs were incubated for 24 h and then measured together with untreated controls. To measure the NPs’ fluorescence at an excitation of 530 nm and emission of 575 nm, a microplate reader Spark multimode microplate reader (Tecan, Switzerland) was used. Immediately after this measurement, images of the cells were made on an IX71 fluorescence microscope (Olympus) equipped with a DP74 color camera (Olympus) and a 20× lens.

##### Cytotoxicity Test

For cytotoxicity evaluation, SAOS‐2 cells were seeded in 96‐well plates at a density of 10 000 cells cm^−2^ and allowed to adhere for 24 h in the standard growth medium. Following this preincubation, the cells were gently washed with phosphate‐buffered saline (PBS; Thermo Fisher Scientific‐Gibco), and fresh medium containing DiI@PAA‐CeNPs was applied. The NPs were dispersed in either FBS‐supplemented (5%) or FBS‐free Dulbecco's Modified Eagle's Medium (DMEM; Thermo Fisher Scientific‐Gibco) and incubated with the cells for 6 h. To mitigate nutrient deprivation effects, FBS was subsequently added to all wells to reach a final concentration of 5%, and the cells were incubated for an additional 18 h. After incubation with the NPs, the medium was removed and replaced with a fresh one containing 10% MTS reagent (CellTiter 96 AQueous One Solution Cell Proliferation Assay; Promega). Cells were incubated for an additional 2 h in a humidified incubator with 5% CO_2_ at 37 °C, and the resulting metabolic activity—indicative of cell viability—was measured by recording absorbance at 492 nm using a microplate reader Spark multimode microplate reader (Tecan, Switzerland), with background correction at 620 nm. To determine the cell number, the total DNA content of living cells was quantified using the CyQUANT NF Cell Proliferation Assay Kit (Thermo Fisher Scientific ‐ Invitrogen). After MTS measurement, the cells were washed with PBS, incubated with the CyQUANT staining solution according to the manufacturer's instructions, and the fluorescence signal was measured at an excitation of 485 nm and emission of 530 nm using a bottom‐reading configuration (4 × 4) on the same microplate reader. Experimental results were expressed as percentages relative to untreated control cells (set as 100%). Statistical analysis was conducted using Statistica software. All data represent the mean ± standard deviation from at least three independent experiments, each performed in six technical replicates.

##### ICP‐MS Measurement

Cerium concentrations were determined using ICP‐MS as reported previously.^[^ ^45^ ^]^ Cells were incubated with an equivalent concentration of PAA‐CeNPs or DiI@PAA‐CeNPs for 24 h. Following incubation, the cells were harvested, thoroughly washed with PBS to remove extracellular particles, and subsequently lysed for Ce analysis. Samples were digested in aqua regia (HCl + HNO_3_) for 24 h and subsequently diluted with 2% v/v HNO_3_ prior to analysis. Calibration curves were prepared using a blank and a multielement rare earth standard (Analytika Ltd, Czech Republic). Measurements were performed on a Thermo iCAP‐Q system (Thermo Bremen, Germany) equipped with a Peltier‐cooled spray chamber (2 °C) and Meinhard‐type nebulizer. Each sample was measured in triplicate, and the average value was reported. Three independent biological replicates were measured for each sample, and the average value was reported. The Ce content was normalized to cell number.

##### Lysosome Staining

Lysosomes were stained using lysosome‐specific probe NIF,^[^ ^71^, ^72^ ^]^ which is available—along with probes of differing selectivity and properties—through scientific collaboration with Dr. Martin Havlík (Department of Analytical Chemistry, UCT Prague). SAOS‐2 cells were seeded in a 4‐well glass slide (Thermo Scientific Nunc Lab‐Tek II Chamber Slide, USA) at a density of 20 000 cells cm^−2^ and allowed to adhere for 24 h in the standard growth medium. After washing, 500 μl of the media (with 5% FBS or without FBS) with 100 μg ml^−1^ of DiI@PAA‐CeNPs was added for 4 h. Then 250 nM of NIF dye was added and incubation was performed for 10 min in the humidified incubator with 5% CO_2_ at 37 °C. After incubation, the cells were washed with PBS, and images were made on an IX71 fluorescence microscope (Olympus) equipped with a DP74 color camera (Olympus) and a 100× lens.

##### Mitochondria Staining

The SAOS‐2 cells were seeded as previously described for the lysosomal staining. After washing, 500 μl of the media (with 5% FBS or without FBS) with 100 μg ml^−1^ of DiI@PAA‐CeNPs was added for 4 h. Then after washing with fresh medium, the cells were stained with 100 nM MitoTracker Green (Thermo Fisher Scientific, USA) at 37 °C for 30 min, then washed with PBS and images were made on IX71 fluorescence microscope (Olympus) equipped with a DP74 color camera (Olympus) and 100× lens.

##### Cytoskeleton and Nuclei Staining

The SAOS‐2 cells were seeded as previously described for the lysosomal staining. After washing, 500 μl of the media (with 5% FBS) with 100 μg ml^−1^ of DiI@PAA‐CeNPs was added for 24 h. Then, the cells were washed, fixed with 4% paraformaldehyde for 15 min at room temperature, and permeabilized with 0.1% Triton X‐100 for 20 min. Following another PBS wash, a blocking was performed with 1% FBS + 0.05% Tween at room temperature for 30 min. Cells were then stained with fluorescence dyes: actin filaments with Phalloidin‐Alexa Fluor 488 in blocking solution at 37 °C in the dark for 1 h (1:500; Life Technologies, USA), followed by nuclei staining with 4′,6‐diamidino‐2‐phenylindole (DAPI; Sigma–Aldrich, USA) in PBS at 37 °C for 15 min. Following PBS washes, the cells were air‐dried and mounted with Shandon Immu‐Mount (Thermo Fisher Scientific, USA) for fluorescent microscopy. Confocal images of the cells were acquired using a Leica SP8X microscope (Leica Microsystems, Germany) equipped with a confocal scanning head, a Leica DFC365 FX monochrome digital CCD camera, an HC PL APO CS2 63×/1.40 OIL objective, a 405 nm excitation laser, and a white light laser (WLL; EX 579/EM 599; hybrid detector) (Leica Microsystems, Germany). The images were rendered using the LasX software (Leica Microsystems, Germany).

##### TEM

SAOS‐2 cells were seeded on round cover glass (*d* = 12 mm) placed in a 24‐well plate at a density of 15 000 cells cm^−2^ and allowed to adhere for 24 h in the standard growth medium. After washing with PBS, the standard growth medium was replaced with DMEM medium with 100 μg ml^−1^ of both CeNPs and incubated for 4 h. After washing with PBS, the cells were fixed with 1% glutaraldehyde and 2.5% paraformaldehyde in 0.1 M Sörensen's sodium‐potassium phosphate buffer (SB) at pH 7.2–7.4 (all reagents from Thermo Fisher Scientific) for 1 h at room temperature and then kept on ice for 3 h until next manipulation.

After washing with SB, the cells were fixed with 1% osmium tetroxide for 1 h, dehydrated in a graded series of acetone, and embedded in Epon‐Durcupan. Ultrathin sections of 80 nm were prepared using an ultramicrotome Leica EM UC6 (Leica Microsystems) with a diamond knife (Diatome). The sections were mounted on formvar‐coated 3.05 mm copper slots (Agar Scientific) and examined in a JEOL JEM‐1400 Flash transmission electron microscope operated at 80 kV and equipped with a Matataki Flash CMOS camera (JEOL).

##### FIB‐SEM Lamella Preparation and Characterization

For FIB lamella preparation, SAOS‐2 cells incubated with or without NPs were fixed via chemical fixation, following previously established protocols.^[^ ^45^ ^]^ EM imaging and lamellae fabrication for STEM were performed using a LYRA Tescan dual‐beam system, which combines FIB and SEM capabilities and is equipped with a gas injection system. To minimize radiation damage during milling, regions of interest were coated with protective layers of silicon oxide. Lamellae were prepared using the in situ lift‐out technique, with the FIB operating at 30 kV and the SEM at 5 kV beam energy.^[^ ^52^ ^]^ HR‐STEM was carried out using the same JEOL JEM‐2200FS and an aberration‐corrected JEOL NEOARM 200 F microscopes with a Schottky‐type field emission gun at an accelerating voltage of 200 kV using a HAADF detector as described previously. Corresponding EDX maps were acquired using a JEOL JED‐2300 detector.

## Supporting Information

Supporting Information is available from the Wiley Online Library or from the author.

## Conflict of Interest

The authors declare no conflict of interest.

## Supporting information

Supplementary Material

## Data Availability

The data that support the findings of this study are available from the corresponding author upon reasonable request.
