# Supplementary material for: To Dye or Not to Dye: Unraveling the Impact of Surface Chemistry on Cerium Oxide Nanoparticles–Cell Interactions
Source: Small Sci. 2025 Dec 18;6(2):e202500446. doi: 10.1002/smsc.202500446 (PMC12908425; doi:10.1002/smsc.202500446)
Supplement: Supplementary file 1 — Supplementary Material [file SMSC-6-e202500446-s001.pdf]

Supporting Information for

To Dye or Not to Dye: Unravelling the Impact of Surface Chemistry on Cerium Oxide Nanoparticles–Cell Interactions

Kanika Dulta[1,2], Thu Ngan Dinhová[3], Marie Hubálek Kalbáčová[4*], Xiaohui Ju[1*]

[1]*Department of Chemistry and Biochemistry, Mendel University in Brno, Zemědělská 1, 61300, Brno, Czech Republic*

[2]*Center of Advanced Innovation Technologies, VŠB-Technical University of Ostrava, 70800, Ostrava-Poruba, Czech Republic*

[3]*Department of Surface and Plasma Science, Faculty of Mathematics and Physics, Charles University, v Holešovičkách 2, 18000, Prague, Czech Republic*

[4]*Institute of Pathological Physiology, 1st Faculty of Medicine, Charles University, U Nemocnice 5, 12853, Prague, Czech Republic*

*Corresponding Author e-mail: marie.kalbacova@lf1.cuni.cz; xiaohui.ju@mendelu.cz

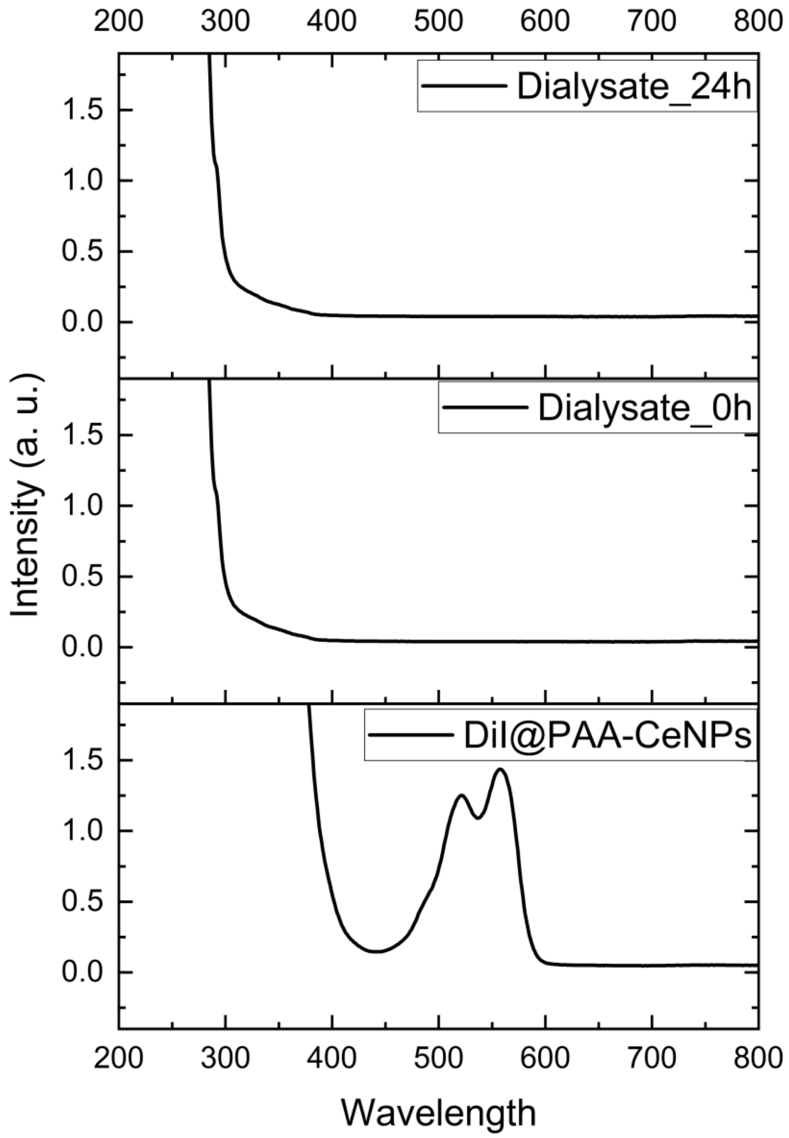

Figure S1: UV–vis absorption spectra of DiI@PAA-CeNPs, 0-hour dialysate, and 24-hours dialysate. The absorption maxima of DiI are evident in the spectrum of DiI@PAA-CeNPs, with no significant absorbance attributable to DiI present in the dialysate spectra, demonstrating efficient encapsulation and negligible dye leakage after 24 hours. Experimental methods were described in the main text.

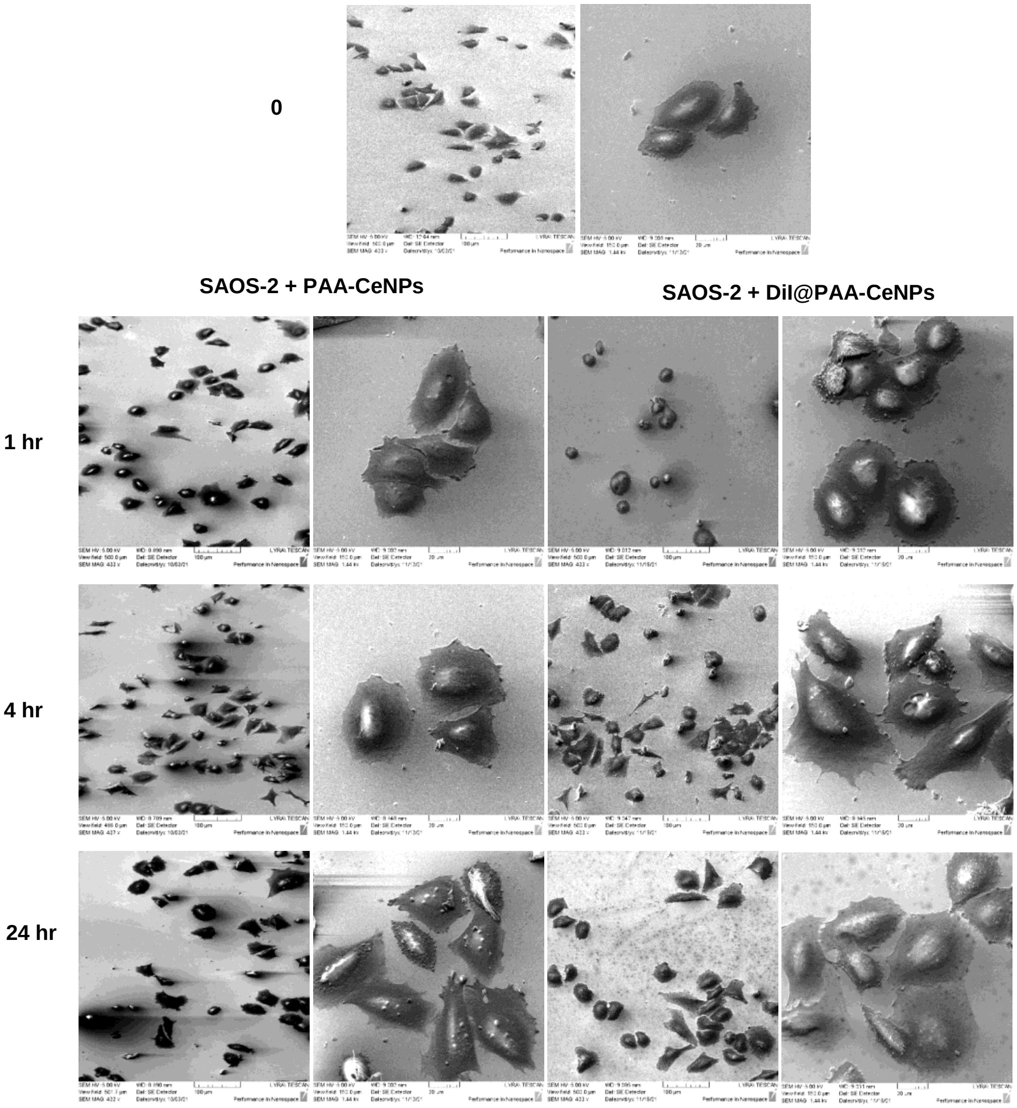

Figure S2. SEM micrographs of SAOS-2 cells co-incubated with PAA-CeNPs and DiI@PAA-CeNPs for 0, 1, 4, and 24 hours. The presence and type of nanoparticles do not induce any observable morphological alterations in the cells. Experimental methods were described in the main text.

Table S1: Composition conditions for zeta potential and hydrodynamic diameter measurement in different solutions. The polydispersity index (PDI) values of both particles in DMEM supplemented with FBS increased significantly compared to those in media without FBS, likely due to the heterogeneous mixture of proteins present in FBS.

Sample	Medium	pH	Polydispersity index (PDI)	Conductivity (mS/cm)	Serum	DiI charge	Notes
**PAA-CeNPs**	Water	6.5	0.2214 ± 0.0058	0.0612	N/A	N/A	Baseline stability
**DiI@PAA-CeNPs**	Water	6.5	0.2531 ± 0.0038	0.0095	N/A	Cationic	ζ shift due to DiI adsorption
**PAA-CeNPs**	DMEM	7.4	0.2587 ± 0.0090	14.27	N/A	N/A	Stability in cell culture medium
**DiI@PAA-CeNPs**	DMEM	7.4	0.2486 ± 0.0079	14.27	N/A	Cationic	Buffer pH where DiI is protonated
**PAA-CeNPs**	DMEM + FBS	7.4	0.5752 ± 0.0923	14.68	5% FBS	N/A	Control at physiological ionic strength
**DiI@PAA-CeNPs**	DMEM + FBS	7.4	0.4917 ± 0.0251	14.68	5% FBS	Cationic	Buffer pH where DiI is protonated

Figure S3. Representative Dynamic Light Scattering (DLS) raw distribution curves of PAA-CeNPs measured in water, shown as intensity-, volume-, and number-weighted profiles. The measurements were performed in triplicate independent runs. The corresponding average hydrodynamic diameter and polydispersity index (PDI) values are summarized in Table S1 (including samples in DMEM and DMEM + FBS).

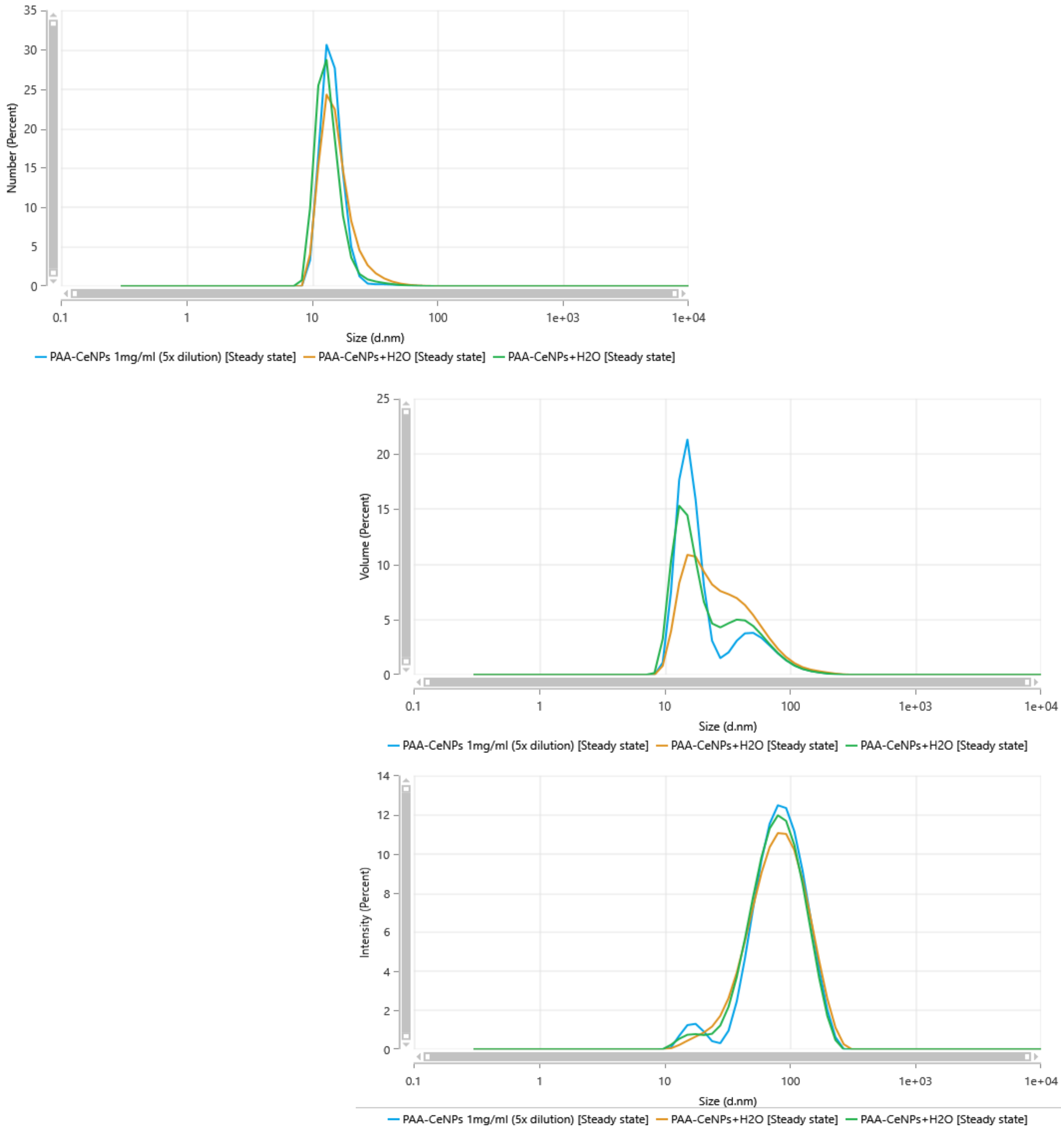

Table S2: Complete chemical composition of Gibco™ DMEM (Low Glucose, Pyruvate, No Glutamine, No Phenol Red) formulations from Thermo Fischer (Catalog number 11054020).

Component	Molecular Weight (g/mol)	Concentration (mg/L)	Concentration (mM)
**Amino Acids**			
Glycine	75.0	30.0	0.4
L-Arginine hydrochloride	211.0	84.0	0.398
L-Cystine 2HCl	313.0	63.0	0.201
L-Histidine hydrochloride-H_2O	210.0	42.0	0.2
L-Isoleucine	131.0	105.0	0.802
L-Leucine	131.0	105.0	0.802
L-Lysine hydrochloride	183.0	146.0	0.798
L-Methionine	149.0	30.0	0.201
L-Phenylalanine	165.0	66.0	0.4
L-Serine	105.0	42.0	0.4
L-Threonine	119.0	95.0	0.798
L-Tryptophan	204.0	16.0	0.078
L-Tyrosine disodium salt dihydrate	261.0	104.0	0.398
L-Valine	117.0	94.0	0.803
**Vitamins**			
Choline chloride	140.0	4.0	0.029
D-Calcium pantothenate	477.0	4.0	0.008
Folic Acid	441.0	4.0	0.009
Niacinamide	122.0	4.0	0.033
Pyridoxine hydrochloride	206.0	4.0	0.019
Riboflavin	376.0	0.4	0.001
Thiamine hydrochloride	337.0	4.0	0.012
i-Inositol	180.0	7.2	0.04
**Inorganic Salts**			
Calcium Chloride ($CaCl_2$) (anhyd.)	111.0	200.0	1.802
Ferric Nitrate ($Fe(NO_3)_3 \cdot 9H_2O$)	404.0	0.1	0.00025
Magnesium Sulfate ($MgSO_4$) (anhyd.)	120.0	97.67	0.814
Potassium Chloride (KCl)	75.0	400.0	5.333
Sodium Bicarbonate ($NaHCO_3$)	84.0	3700.0	44.048
Sodium Chloride (NaCl)	58.0	6400.0	110.345
Sodium Phosphate monobasic ($NaH_2PO_4 \cdot H_2O$)	138.0	125.0	0.906
**Other Components**			
D-Glucose (Dextrose)	180.0	1000.0	5.556
Sodium Pyruvate	110.0	110.0	1.0
